# Co-clustering of EphB6 and ephrinB1 in trans restrains cancer cell invasion

**DOI:** 10.1038/s42003-024-06118-4

**Published:** 2024-04-16

**Authors:** Lung-Yu Liang, Niall D. Geoghegan, Michael Mlodzianoski, Andrew Leis, Lachlan W. Whitehead, Minglyanna G. Surudoi, Samuel N. Young, Peter Janes, Doulin Shepherd, Debnath Ghosal, Kelly L. Rogers, James M. Murphy, Isabelle S. Lucet

**Affiliations:** 1 Walter and Eliza Hall Institute for Medical Research, 1G Royal Parade, Parkville, VIC 3052 Australia; 2 Department of Medical Biology, University of Melbourne, 1G Royal Parade, Parkville, VIC 3052 Australia; 3 Olivia Newton-John Cancer Research Institute and La Trobe School of Cancer Medicine, Level 5, ONJ Centre, 145 Studley Rd, Heidelberg, VIC 3084 Australia; 4 Department of Biochemistry and Pharmacology, Bio21 Molecular Science and Biotechnology Institute, University of Melbourne, Parkville, VIC 3052 Australia; 5 ARC Centre for Cryo-electron Microscopy of Membrane Proteins, Bio21 Molecular Science and Biotechnology Institute, University of Melbourne, Parkville, VIC 3052 Australia; 6 Drug Discovery Biology, Monash Institute of Pharmaceutical Sciences, Monash University, Parkville, VIC 3052 Australia

**Keywords:** Cell biology, Cellular imaging

## Abstract

EphB6 is an understudied ephrin receptor tyrosine pseudokinase that is downregulated in multiple types of metastatic cancers. Unlike its kinase-active counterparts which autophosphorylate and transmit signals upon intercellular interaction, little is known about how EphB6 functions in the absence of intrinsic kinase activity. Here, we unveil a molecular mechanism of cell-cell interaction driven by EphB6. We identify ephrinB1 as a cognate ligand of EphB6 and show that in trans interaction of EphB6 with ephrinB1 on neighboring cells leads to the formation of large co-clusters at the plasma membrane. These co-clusters exhibit a decreased propensity towards endocytosis, suggesting a unique characteristic for this type of cell-cell interaction. Using lattice light-sheet microscopy, 3D structured illumination microscopy and cryo-electron tomography techniques, we show that co-clustering of EphB6 and ephrinB1 promotes the formation of double-membrane tubular structures between cells. Importantly, we also demonstrate that these intercellular structures stabilize cell–cell adhesion, leading to a reduction in the invasive behavior of cancer cells. Our findings rationalize a role for EphB6 pseudokinase as a tumor suppressor when interacting with its ligands in trans.

## Abstract (web summary)

This study demonstrates that co-clustering of the EphB6 receptor tyrosine pseudokinase with its ephrinB1 ligand on neighboring cells promotes formation of intercellular tubules. This stabilizes cell-cell adhesion and reduces cancer cell invasion.

## Introduction

The receptor tyrosine kinase (RTK) family plays fundamental roles in cell proliferation, differentiation, and migration, shaping the complexity of multicellular organisms by transducing extracellular stimulation to intracellular signals^1^. Among the 58 human RTKs, 14 members belong to the erythropoietin-producing human hepatocellular (Eph) receptor family. Unlike other RTKs whose ligands are secreted into the extracellular environment in a soluble form, the ligands of Eph receptors, called ephrins, are membrane-bound. Therefore, the interaction between Eph receptors and ephrins occurs at sites of cell-to-cell contacts. Upon binding of ephrins to Eph receptors, the receptors form higher order clusters, and these clusters are thought to function as signaling hubs to amplify intracellular phosphorylation and propagate downstream signaling^2,3^. Aberrant expression of Eph receptors is linked to metastasis and multiple types of malignancies. Interestingly, the same Eph receptor can play both tumor-promoting and suppressive roles depending on cellular contexts^4^. To date, no therapeutics against Eph receptor-associated malignancies have been clinically approved^5–7^. This is most likely due to a lack of comprehensive understanding of the activation mechanisms of Eph receptors, the crosstalk among Eph receptors and their membrane-bound ligands, and the complexity of their dichotomous functions.

Eph receptors comprise extracellular and transmembrane domains, as well as an intracellular portion consisting of the juxtamembrane region, a kinase or pseudokinase domain, a sterile-alpha motif (SAM) domain, and a C-terminal PDZ-binding motif. While the kinase activity of Eph receptors is considered critical for signal transduction^3^, each member of the Eph receptor family has at least one splicing isoform where the intracellular kinase domain is truncated, suggesting that the non-catalytic functions of Eph receptors are also important for their functionality^2^. Additionally, two members of the Eph receptor family, EphA10 and EphB6, are classified as pseudokinases, owing to the presence of a pseudokinase domain devoid of catalytic activity within their intracellular portions^2,8^.

The precise mechanisms by which the EphA10 and EphB6 pseudokinases contribute to specific cellular functions and signaling processes remain poorly understood. EphA10 is typically only expressed in normal testes^9^ and is postulated to act as an oncogene, with its expression upregulated in many types of tumors, including breast, prostate, oral, and lung malignancies^10–13^. The role of EphB6 in cancer is still a matter of debate^14–16^, and even though the underlying mechanism through which EphB6 influences cancer progression remains unclear, the existing body of evidence leans toward its role in suppressing metastasis^16–20^. The ephrin ligands of EphB6 remain largely unexplored, but available evidence supports ephrinB1 as a physiological ligand of EphB6^21,22^. EphrinB1 expression is abundant in germinal centers within resident B cells, which selectively repulsed EphB6-expressing follicular helper T cells^23^. While these observations suggest a context-specific repulsion, more typically, ephrinB1 presentation in trans is thought to activate signaling EphB6 pathways, such as in a chromaffin cell line model where EphB6 was activated by ephrinB1, but not ephrinB2^24^. In pathological contexts, an inverse correlation between EphB6 and ephrinB1 protein expression has been reported in metastatic tumors^14,16–18,25^, implying that interaction of EphB6 and ephrinB1 could potentially control tumorigenesis.

In this study, we sought to determine whether ephrinB1 can directly bind to EphB6 and to examine the biological consequences of EphB6 interacting with ephrinB1 at the molecular level. To achieve this, we developed a co-culture system in which fluorescently labeled EphB6 and ephrinB1 were expressed in different sublines of the MDA-MB-231 invasive breast cancer cell line. Using live cell imaging, we demonstrate that EphB6 clusters at the plasma membrane upon interaction with ephrinB1 across cell–cell junctions (in trans), confirming ephrinB1 as a cognate ligand for EphB6. We identify two key oligomerization interfaces in the ligand-binding and cysteine-rich domains of the EphB6 ectodomain that are critical for clustering of ephrinB1-bound EphB6. Interestingly, we demonstrate that EphB6 clusters persist at the plasma membrane and exhibit a reduced propensity towards endocytosis compared to clusters formed by the kinase-active Eph receptor, EphB1. Furthermore, we demonstrate that EphB6:ephrinB1 co-clusters promote the formation of tubular structures between EphB6- and ephrinB1-expressing cells via the engulfment of membrane protrusions, revealing a previously unreported mode of intercellular interaction. Lastly, we conducted three-dimensional tumor spheroid assays, which demonstrated that co-clustering of EphB6 and ephrinB1 decreases the invasiveness of the breast cancer cell line, MDA-MB-231. Taken together, our findings suggest that the absence of intrinsic catalytic activity of EphB6 may be essential for maintaining intercellular homeostasis, which differentiates it from typical RTKs that rely on kinase activity to transduce signals.

## Results

### EphB6 and ephrinB1 co-cluster at the plasma membrane in trans

**Fig. 1:**
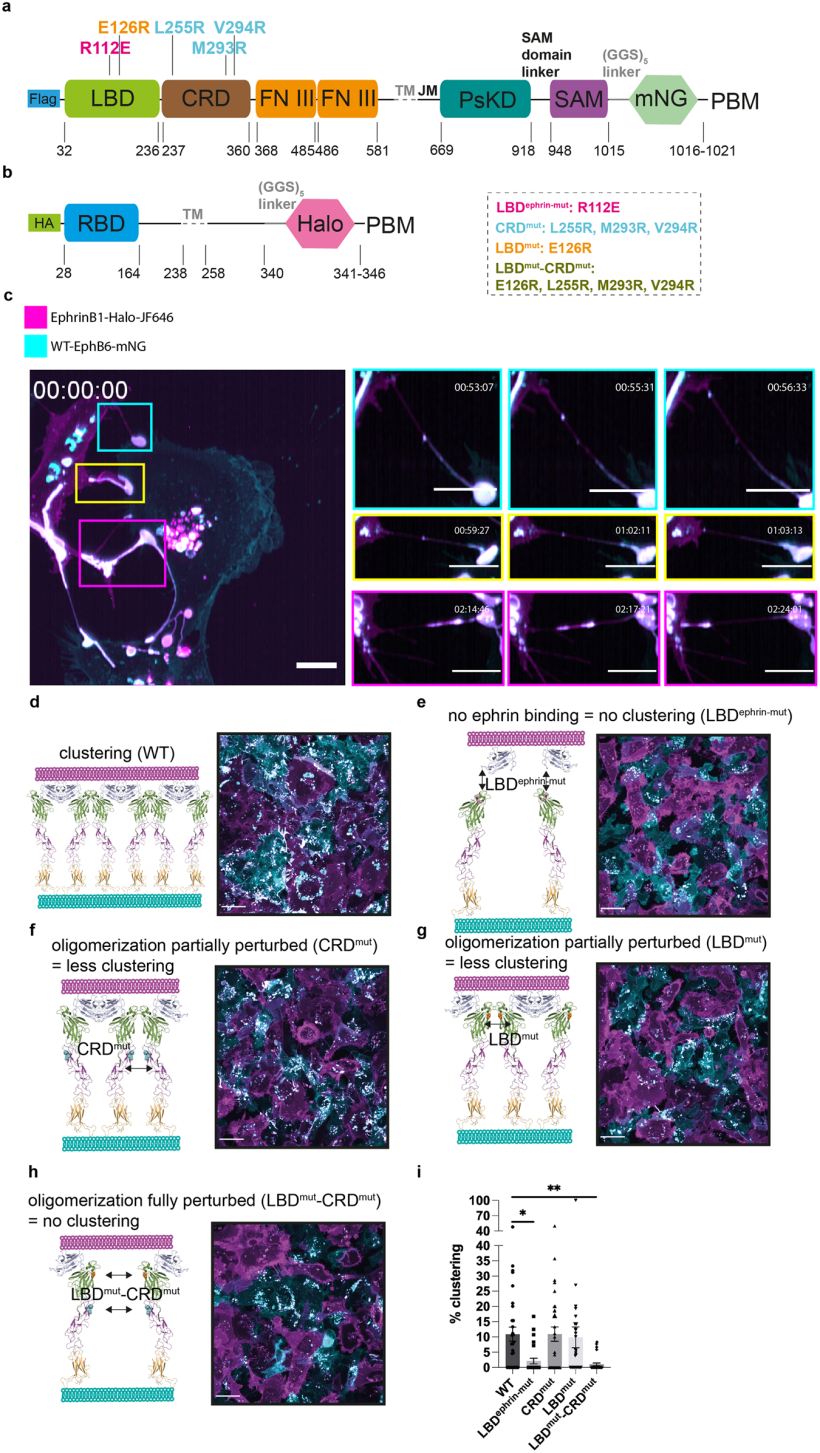
Clustering of EphB6 at the plasma membrane is dependent on ephrinB1 binding and EphB6 oligomerization interfaces. **a** EphB6-mNG wild-type and mutant constructs. R112E (on the putative ephrin binding groove), E126R (on the LBD oligomerization interface), L255R, M293R, and V294R (on the CRD oligomerization interface). Flag: FLAG tag. LBD: ligand-binding domain. CRD: cysteine-rich domain. FN III: fibronectin III domain. TM: transmembrane domain. JM: juxtamembrane region. PsKD: pseudokinase domain. SAM: sterile α-motif. mNG: mNeonGreen. PBM: PDZ domain-binding motif. **b** Wild-type ephrinB1-Halo construct. HA: Hemagglutinin tag. RBD: receptor-binding domain. TM: transmembrane domain. Halo: HaloTag. PBM: PDZ domain-binding motif. **c** Live cell imaging of WT-EphB6-mNG-expressing cells (in cyan) and ephrinB1-Halo-expressing cells (visualized by addition of 50 nM JF646, in magenta) by lattice light-sheet microscopy. The large white patches at the cell junctions are the EphB6:ephrinB1 co-clusters. The EphB6:ephrinB1 co-clusters that are moving along the tubular structures and internalized into the ephrinB1-expressing cells are highlighted. Images are presented as maximum-intensity projections. The scale bar indicates 10 µm. Live cell imaging of **d** WT-EphB6-mNG, **e** LBD^ephrin-mut^-EphB6-mNG, **f** CRD^mut^-EphB6-mNG, **g** LBD^mut^-EphB6-mNG and **h** LBD^mut^-CRD^mut^-EphB6-mNG-expressing MDA-MB-231 cells (in cyan) co-cultured with ephrinB1-Halo-expressing MDA-MB-231 cells (visualized by addition of 50 nM JF646, in magenta) by confocal microscopy. The large white patches at the cell junctions indicate the EphB6:ephrinB1 co-clusters. Images are presented as maximum-intensity projections. The scale bars indicate 20 µm. **i** Quantification of the co-clustering of EphB6 and ephrinB1 from each co-culture system. The % clustering is defined as the number of tubules with co-clusters connecting EphB6- and ephrinB1-expressing cells versus the total number of protrusions connecting EphB6- and ephrinB1-expressing cells. Each data point was derived from a field of view. The mean ± SEM (standard error of the mean) was calculated from three biological replicates including (WT: 30, LBD^ephrin-mut^: 26, CRD^mut^: 31, LBD^mut^: 30 and LBD^mut^-CRD^mut^: 28) fields of view. *P < 0.05 and **P < 0.01 were calculated by one-way ANOVA followed by a Dunnett’s multiple comparison test.

To determine whether ephrinB1 is a ligand of EphB6, we established a co-culture system for live cell imaging. We tagged the C-terminus of full-length EphB6 with mNeonGreen to generate a construct referred to as WT-EphB6-mNG (Fig. 1a). Similarly, we fused a HaloTag to full-length ephrinB1 to generate a construct referred to as ephrinB1-Halo, allowing for fluorescent labeling of ephrinB1 upon addition of a HaloTag binding fluorescent compound (Fig. 1b). In addition to WT-EphB6-mNG, we designed four mutants to probe the mechanisms of ligand-binding and receptor oligomerization of EphB6 (Fig. 1a and Supplementary Fig. 1a–d). We disrupted ephrin binding by mutating arginine 112 to glutamic acid (R112E) within the ligand-binding domain (LBD) – a residue that is highly conserved in other Eph receptors – to generate the LBD^ephrin-mut^-EphB6-mNG construct (Supplementary Fig. 1a)^26–31^. Additionally, we disrupted the predicted EphB6 oligomerization interfaces by introducing mutations into two interfaces that have been previously characterized in the oligomerization of other Eph receptors^26–31^. Three mutations (L255R, M293R, and V294R) were introduced into the cysteine-rich domain (CRD), to generate a construct termed CRD^mut^-EphB6-mNG; one mutation (E126R) was introduced into the LBD domain (LBD^mut^-EphB6-mNG); finally, we combined the LBD mutant (E126R) with the triple CRD mutant (L255R, M293R, and V294R) to generate a construct termed LBD^mut^-CRD^mut^-EphB6-mNG (Supplementary Fig. 1b–d).

All EphB6 constructs were expressed in the MDA-MB-231 invasive breast cancer cell line, which does not endogenously express EphB6^17,19^. EphrinB1-Halo was expressed in parallel in MDA-MB-231 cells to minimize heterogeneity when co-culturing the EphB6-mNG- and ephrinB1-Halo-expressing MDA-MB-231 cells in subsequent experiments. EphB6 and ephrinB1 expression was induced using doxycycline (Supplementary Fig. 1e–g), and the protein expression levels of different EphB6 variants were comparable (Supplementary Fig. 1h). Both EphB6 and ephrinB1 localize to the plasma membrane (Supplementary Fig. 1i, j), consistent with the tagging strategy not impacting localization. We found that co-culturing WT-EphB6-mNG- and ephrinB1-Halo-expressing MDA-MB-231 cells lead to extensive co-clustering of EphB6 and ephrinB1 at the cell–cell interface (Fig. 1c and Supplementary Movie 1). Interestingly, we also observed internalization of smaller EphB6:ephrinB1 clusters in the ephrinB1-expressing cells (Fig. 1c and Supplementary Movie 1), but rarely in the opposite direction. A previous study showed that the kinase-inactive EphB2 mutant promotes internalization of the EphB2:ephrinB1 clusters into ephrinB1-expressing cells^32^. As EphB6 lacks kinase activity, our observation is consistent with the notion that kinase activity of the Eph receptors dictates the direction of Eph:ephrin cluster internalization^33^. Collectively. these data confirm ephrinB1 as a cognate ligand of EphB6 and illustrate that EphB6, despite lacking catalytic activity, retains a capacity for clustering akin to kinase-active Eph receptors^34,35^.

To pinpoint the molecular determinants governing co-clustering of EphB6 and ephrinB1, we performed quantitative light microscopy analysis across various EphB6 variants. In contrast to WT-EphB6-mNG (Fig. 1c, d), LBD^ephrin-mut^-EphB6-mNG mutant did not cluster when co-cultured with cells expressing ephrinB1-Halo (Fig. 1e, i). The absence of clusters is consistent with the R112E mutation impairing ligand binding, which indicates an essential role for the interaction of EphB6 and ephrinB1 in cluster formation. In addition, co-culture of ephrinB1-Halo-expressing cells with CRD^mut^-EphB6-mNG- and LBD^mut^-EphB6-mNG-expressing cells resulted in less clustering, while no clustering was observed with LBD^mut^-CRD^mut^-EphB6-mNG-expressing cells (Fig. 1f, i). This indicates that clustering of ephrinB1-bound EphB6 is mediated by the two EphB6 extracellular oligomerization interfaces. Perturbation of both oligomerization interfaces fully abolishes the ability of EphB6 to cluster, even in the presence of ephrinB1 (Fig. 1f, i).

### EphB6:ephrinB1 co-clusters exhibit a decreased propensity to undergo endocytosis in EphB6-expressing cells

**Fig. 2:**
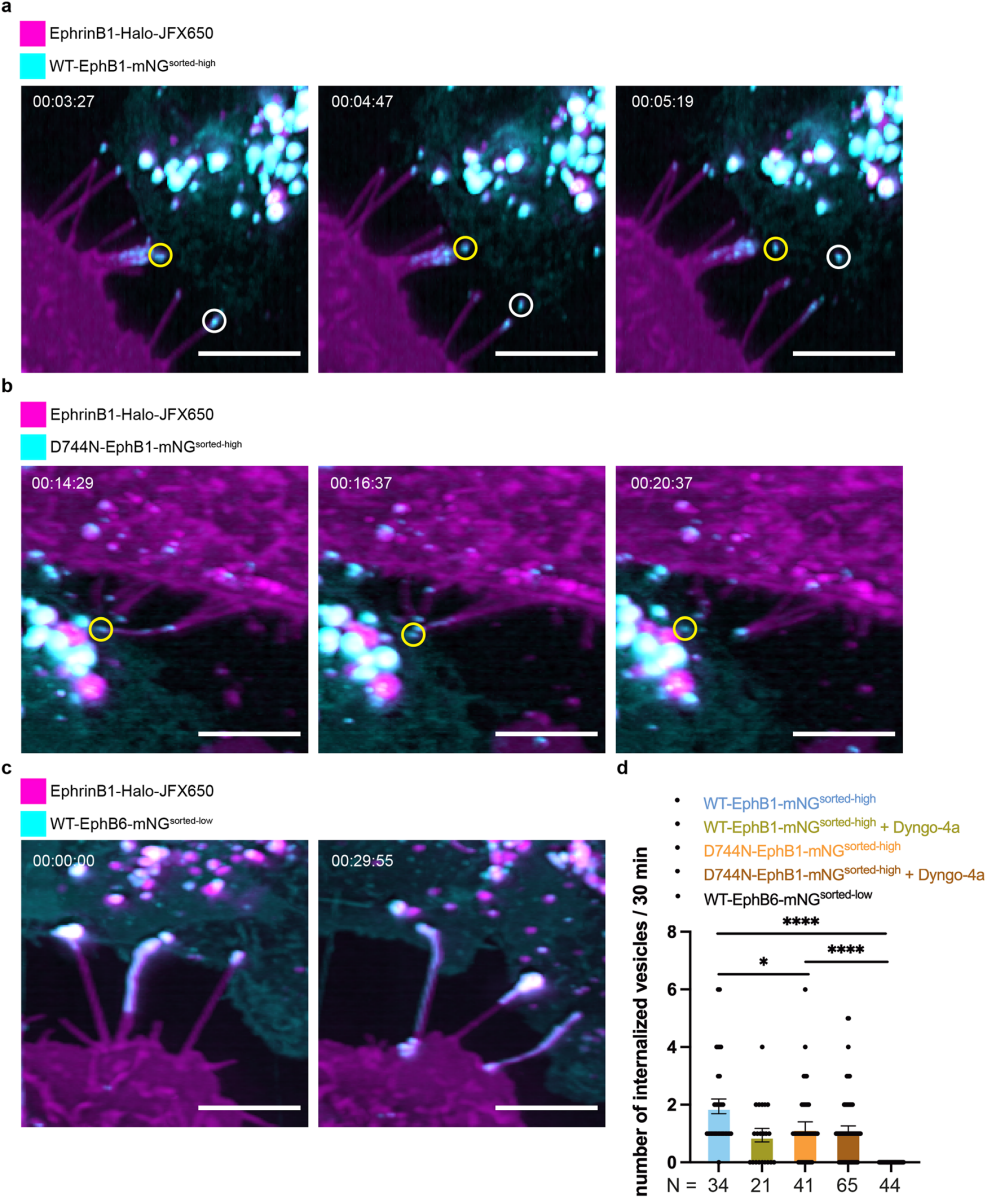
EphB6:ephrinB1 co-clusters are more resistant to endocytosis. Live cell imaging of **a** WT-EphB1-mNG-, **b** D744N-EphB1-mNG- and **c** WT-EphB6-mNG-expressing MDA-MB-231 cells (in cyan) co-cultured with ephrinB1-Halo-expressing MDA-MB-231 cells (visualized by addition of 50 nM JFX650, in magenta) by lattice light-sheet microscopy. The circles indicate the clusters tracked along the time course. Images are presented as maximum-intensity projections. The scale bars indicate 10 µm. **d** Quantification of the internalization rate of the clusters. *N* = the number of membrane protrusions with clusters that were counted to measure the cluster internalization rate, from two to three biologically independent experiments. The data are represented by mean ± SEM (standard error of the mean). *P < 0.05 and ****P < 0.0001 were calculated by one-way ANOVA followed by a Dunnett’s multiple comparison test.

Ligand-bound RTKs typically undergo endocytosis as a mechanism to terminate signal transduction^36^. Bidirectional endocytosis of the clustered ephrin-bound kinase-active Eph receptors have been broadly reported^34,35,37,38^. In contrast, our live cell imaging experiments revealed that EphB6:ephrinB1 co-clusters persist at the cell–cell interface and rarely undergo endocytosis into the EphB6-expressing cells. This observation prompted us to investigate whether the lack of kinase activity for EphB6 reduces its propensity to undergo endocytosis when bound to ephrinB1. To test this hypothesis, we performed additional live cell imaging experiments using the catalytically active Eph receptor kinase, EphB1 that also binds ephrinB1^39^. We generated both wild-type mNeonGreen tagged full-length EphB1 (referred to as WT-EphB1-mNG), as well as a kinase-inactive mutant by mutating the catalytic Asp in the HRD motif in the catalytic loop of the kinase domain (referred to as D744N-EphB1-mNG, (Fig. 2 and Supplementary Fig. 2a). These constructs were stably introduced into MDA-MB-231 cells (Supplementary Fig. 2b–f). We then co-cultured the cells expressing WT-EphB1-mNG or D744N-EphB1-mNG with ephrinB1-Halo-expressing cells. Both WT-EphB1-mNG and D744N-EphB1-mNG form clusters upon interaction with ephrinB1-Halo, similar to those observed with WT-EphB6-mNG-expressing cells, but the clusters are relatively smaller and at the tip of the membrane protrusions of ephrinB1-Halo-expressing cells (Fig. 2a–c, Supplementary Fig. 2g–i, Supplementary Movies 2–4). As expected, we observed constant endocytosis of the EphB1:ephrinB1 clusters into the EphB1-expressing cells (Fig. 2a, b, Supplementary Movies 2–3), consistent with observations with EphA2:ephrinA1 and EphB2:ephrinB1 pairs in other studies^34,35^. Quantification of the number of EphB1:ephrinB1 co-clusters being endocytosed in the EphB1-expressing cells over a 30-min time frame showed that the kinase-active WT-EphB1 exhibits a two-fold higher rate of endocytosis than the kinase-inactive EphB1 mutant (Fig. 2d). Furthermore, the endocytosis rate of kinase-active WT-EphB1, but not that of the kinase-inactive mutant, could be hindered in the presence of a dynamin inhibitor, Dyngo-4a. (Fig. 2d). Together, these data suggest that the turnover of EphB1:ephrinB1 clusters is partially governed by EphB1 kinase activity and clathrin-mediated endocytosis. Strikingly, we did not observe any endocytosis of the EphB6:ephrinB1 co-clusters over the same time frame (Fig. 2c, d and Supplementary Movie 4). Internalization of EphB6:ephrinB1 co-clusters into EphB6-expressing cells was only captured by longer time-lapse image acquisitions (Supplementary Movie 5). However, such internalization in bulk was inconsistent with clathrin-mediated endocytosis, by which three to nine membrane proteins are typically packaged into a vesicle^40^. Although we cannot rule out if overexpression of either protein is responsible for this observation (Supplementary Fig. 2b–f), our data suggest that EphB6:ephrinB1 co-clusters are more resistant to endocytosis and are internalized by a mechanism that is distinct from clathrin-mediated endocytosis.

### Unique tubular structures between cells arise from EphB6:ephrinB1 interaction

**Fig. 3:**
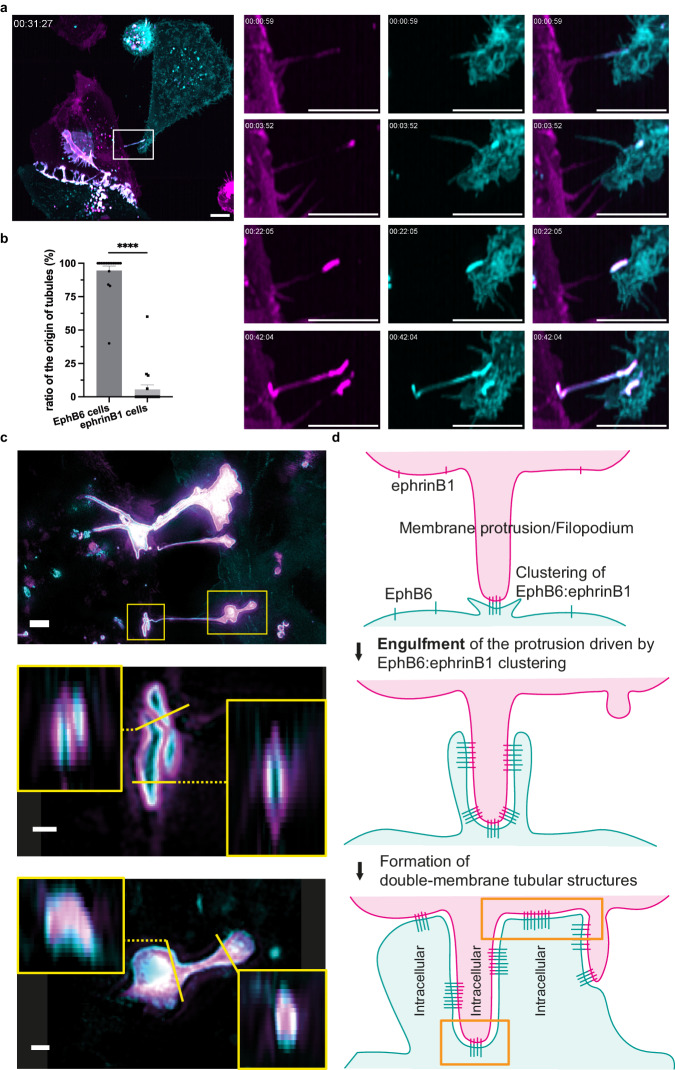
Formation of tubular structures through engulfment of membrane protrusions driven by EphB6:ephrinB1 co-clusters. **a** Live cell imaging of WT-EphB6-mNG-expressing MDA-MB-231 cells (cyan) and ephrinB1-Halo-expressing cells (magenta, visualized by addition of 50 nM JF646) by lattice light-sheet microscopy. Many tubular structures decorated by the EphB6:ephrinB1 co-clusters (in white) are seen in the time-lapse images. The white box highlights a newly formed tubular structure within the EphB6:ephrinB1 co-clusters. Images are presented as maximum-intensity projections. The scale bar indicates 10 µm. **b** Quantification of the origin of tubule formation in which EphB6:ephrinB1 co-clusters are present. Each data point was derived from a field of view. The data are represented by mean ± SEM (standard error of the mean) from three biological replicates containing a total of 16 fields of view. ****P < 0.0001 were calculated by unpaired and two-tailed Student’s *t-*test. **c** Live cell imaging by three-dimensional structured illumination microscopy (3D SIM) on the tubular structures decorated by the EphB6 (cyan):ephrinB1 (magenta) co-clusters, from a co-culture system of WT-EphB6-mNG-expressing and ephrinB1-Halo-expressing MDA-MB-231 cells. The scale bar in the upper panel indicates 2 µm, and the scale bars in the middle and lower panels indicate 0.5 µm. **d** Proposed mechanism for formation of tubular structures decorated by the EphB6:ephrinB1 co-clusters. Membrane protrusions/filopodia of the ephrinB1-expressing cells reach the EphB6-expressing cells, leading to clustering of EphB6 and ephrinB1 at the cell junction. Subsequently, the EphB6:ephrinB1 co-clusters extend, causing the plasma membrane of the EphB6-expressing cells to engulf the membrane protrusions of the ephrinB1-expressing cells. This engulfment process gives rise to double-membrane tubular structures where the EphB6:ephrinB1 co-clusters reside. This proposed model is also supported by the quantification results presented in (**b**), showing that more than 90% of the engulfment events are consistent with this model. The orange boxes indicate the tubular junctions that are highlighted in c.

During our live cell imaging experiments, we consistently observed the formation of tubular structures between EphB6- and ephrinB1-expressing cells, which were decorated with EphB6:ephrinB1 co-clusters (Figs. 1c, d, f, g, 2c, 3a and Supplementary Movie 6). We noticed that the co-cultured cells with higher confluency had more frequent cell–cell contact, resulting in the formation of more tubular structures decorated by the EphB6:ephrinB1 co-clusters (Fig. 1d). Interestingly, quantitative analysis of our lattice light-sheet microscopy data showed a highly unidirectional formation of the tubules with co-clusters – EphB6:ephrinB1 clusters first form when membrane protrusions of ephrinB1-expressing cells are in contact with EphB6-expressing cells (Fig. 3a, b and Supplementary Movie 6). Subsequently, EphB6:ephrinB1 co-clusters are established at discrete sites along the membrane protrusions. Concomitantly, a tubular structure is formed between the two connected cells bearing multiple EphB6:ephrinB1 co-clusters (Fig. 3a and Supplementary Movie 6). The formation of these tubular structures appears to be intrinsic to EphB6:ephrinB1 clustering and independent of the cell lines involved, as similar structures are also observed between HEK293 cells expressing untagged ephrinB1 and EphB6-expressing MDA-MB-231 cells (Supplementary Movie 7). These tubular structures typically exhibit a stronger plasma membrane signal compared to typical membrane protrusions or tubes that occurred outside of the EphB6:ephrinB1 co-clusters (Supplementary Fig. 3a–d), consistent with EphB6:ephrinB1 co-clusters contributing to the formation of thicker tubular structures that connect cells.

To further characterize these tubular structures, we next applied three-dimensional structured illumination microscopy (3D SIM). Our 3D SIM data showed that these tubular structures possess a double-membrane morphology, originating from the plasma membrane of both the receptor- and ligand-expressing cells (Fig. 3c and Supplementary Fig. 3e). The junctions of the tubules at EphB6- and ephrinB1-expressing cells are both closed-ended (Fig. 3c and Supplementary Fig. 3e). To our knowledge, the double-membrane organization observed for these types of intercellular connections has not been reported previously. Substantiated by quantitative analysis showing a unidirectional formation of tubules with co-clusters (Fig. 3b), our 3D SIM data can be explained by our proposed engulfment model (Fig. 3b) in which the membrane protrusions of the ephrinB1-expressing cells are engulfed by EphB6-expressing cells, following the heterotypic interaction of EphB6 and ephrinB1 (Fig. 3a–c). The engulfed plasma membrane of ephrinB1-expressing cells forms the inner layer of the tubular structures adjacent to the EphB6-expressing cells. This yields tubular structures, which are re-enforced by a double-membrane and surrounded by a cytoplasmic environment (Fig. 3c, d).

### Visualization of tubular structures bearing the EphB6:ephrinB1 co-clusters using high-resolution cryo-electron tomography

To further validate our engulfment model, we established a workflow combining correlative light and electron microscopy (CLEM) with cryo-electron tomography (cryo-ET). This approach allowed us to first identify tubular structures bearing the EphB6:ephrinB1 co-clusters using light microscopy, followed by cryo-ET imaging (Supplementary Fig. 4). In our CLEM workflow, we cultured the WT-EphB6-mNG- and ephrinB1-Halo-expressing MDA-MB-231 cells on EM grids and fixed the cells using glutaraldehyde and paraformaldehyde. Notably, the morphology of the tubular structures remained largely unaffected by the fixation process, as the double-membrane organization retained its integrity and closely resemble that observed in live cells (Fig. 3c and Supplementary Fig. 3e).

**Fig. 4:**
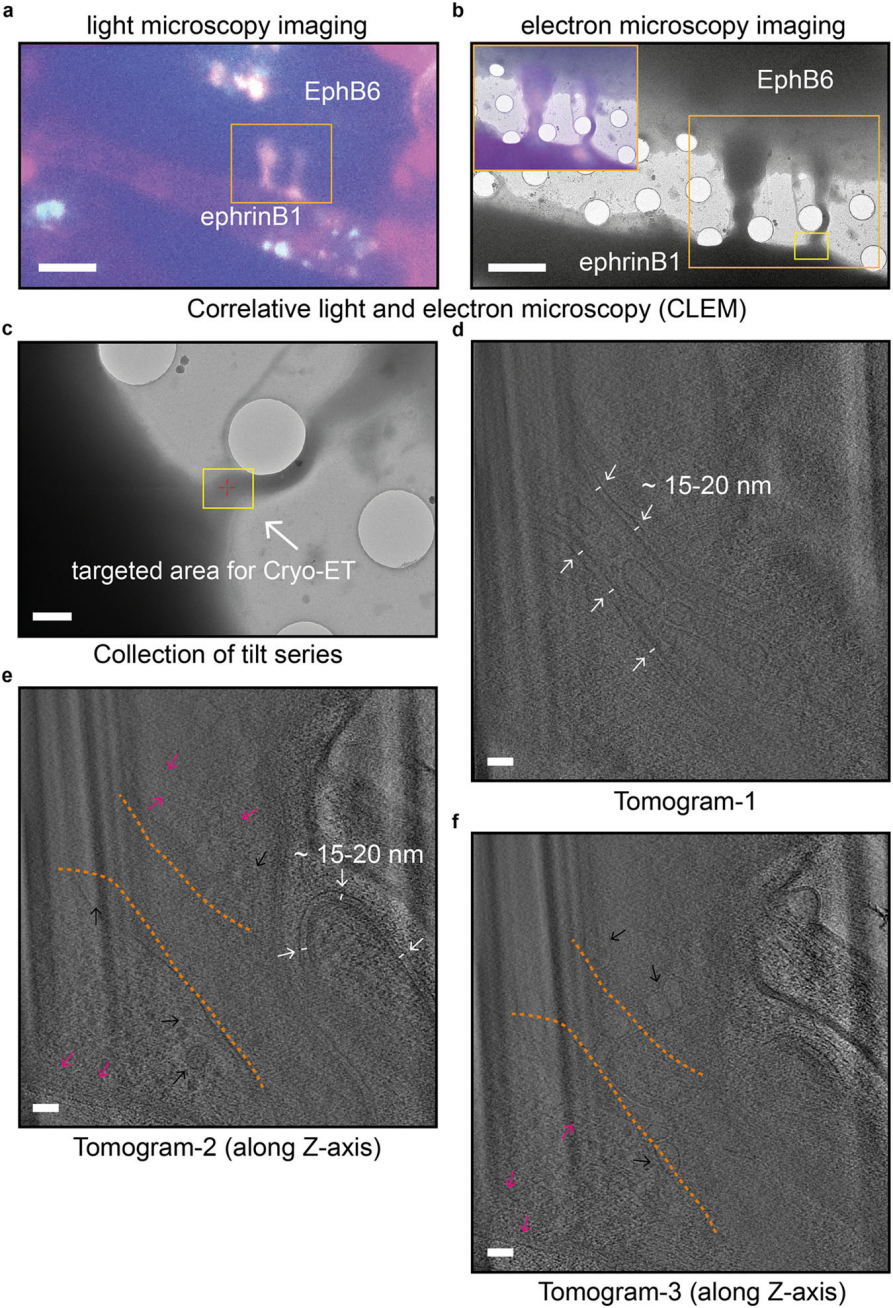
High-resolution cryo-electron tomography of tubular structures decorated by the EphB6:ephrinB1 co-clusters. **a** A light microscopy image of the tubular structures decorated by the EphB6:ephrinB1 co-clusters. The scale bar indicates 10 µm. **b** An electron micrograph of the same tubular structures identified in Panel (**a**). The orange box indicates the CLEM area. The yellow box indicates the area targeted for tilt series collection. The scale bar indicates 5 µm. **c** An intermediate magnification (4800×) micrograph of the tubular structure. The yellow box indicates where cryo-ET data was collected. The scale bar indicates 1 µm. **d** A tomographic slice through the reconstructed tomogram reveals a double-membrane tubular structure, with a spacing of 15–20 nm between the double membranes. The scale bar indicates 100 nm. **e**, **f** Two different slices of the same tomogram (along the Z-axis) reveal the double-membrane tubular structure is surrounded by the cytoplasmic environment, as shown by the presence of vesicles (indicated by the black arrows) and actin filaments (indicated by the magenta arrows). In **e** an enclosed double-membrane protrusion, with a spacing of 15–20 nm between the membranes, is present. This resembles the protrusion structures identified by 3D SIM in Fig. 3c. The scale bar indicates 100 nm. 10 slices were averaged to generate figures. Thickness of each slice shown in **d**–**f** is ~10 nm.

The targeted tubular structures, identified from the light microscopy atlases, exhibited a strong fluorescence signal, always connecting one EphB6-expressing and one ephrinB1-expressing cell (Fig. 4a). Using the coordinates on the Finder grids, we then precisely identified the identical corresponding location in the electron microscopy atlases, enabling the correlation between the light and the electron microscopy atlases. This allowed us to accurately locate the tubular structures harboring the EphB6:ephrinB1 co-clusters by electron microscopy (Fig. 4b) and further examine them using cryo-ET (Fig. 4c). Consistent with our 3D SIM data (Fig. 3c and Supplementary Fig. 3e), the reconstructed tomograms show the presence of a tubular structure with a double-membrane boundary (Fig. 4d–f and Supplementary Movie 8). The double membrane has a consistent gap with a spacing of 15–20 nm (Fig. 4d, e). Such a parallel double-membrane organization with curvature (Fig. 4d, e) is morphologically distinct from microtubules, which have a ~25 nm diameter and are straight in published tomograms^41,42^. Within those tubular structures in our tomograms, intracellular vesicles and filamentous structures resembling the cytoskeletal system are clearly visible (Fig. 4d–f and Supplementary Movie 8). Notably, by navigating along the Z-axis of our tomograms, vesicles and the actin filaments outside the double-membrane tubular structure could also be clearly observed (Fig. 4e, f and Supplementary Movie 8), suggesting that the double-membrane tubular structures are embedded in a cytoplasmic environment.

Although we could not visualize the co-clustered EphB6:ephrinB1 protein complexes within the double-membrane tubular structures from our tomograms, perhaps due to the low contrast in crowded cellular environments, we used structural modeling to speculate on the organization of ephrinB1-ligated EphB6 protein complexes. We hypothesized that the EphB6:ephrinB1 ectodomains would reside in the space between the double membrane and estimated their lengths based on AlphaFold models superimposed on the EphA2:ephrinA5 complex crystal structure (Supplementary Fig. 5). This model suggest that the ectodomains of EphB6 and ephrinB1 span a range of 16–34 nm, depending on the conformation of the linker between the N´-fibronectin III domain and C´-fibronectin III domains as well as the unstructured loops (Supplementary Fig. 5e, f). A previous study demonstrated that the EphA3 ectodomains spanned 17–29 nm in length at the plasma membrane^43^. This spacing is consistent with our cryo-ET data, which showed a 15–20 nm spacing between the double membranes from the tubular structures where EphB6:ephrinB1 co-clusters reside (Fig. 4d, e).

### EphB6:ephrinB1 interaction promotes stable adhesion and reduces cell invasiveness

**Fig. 5:**
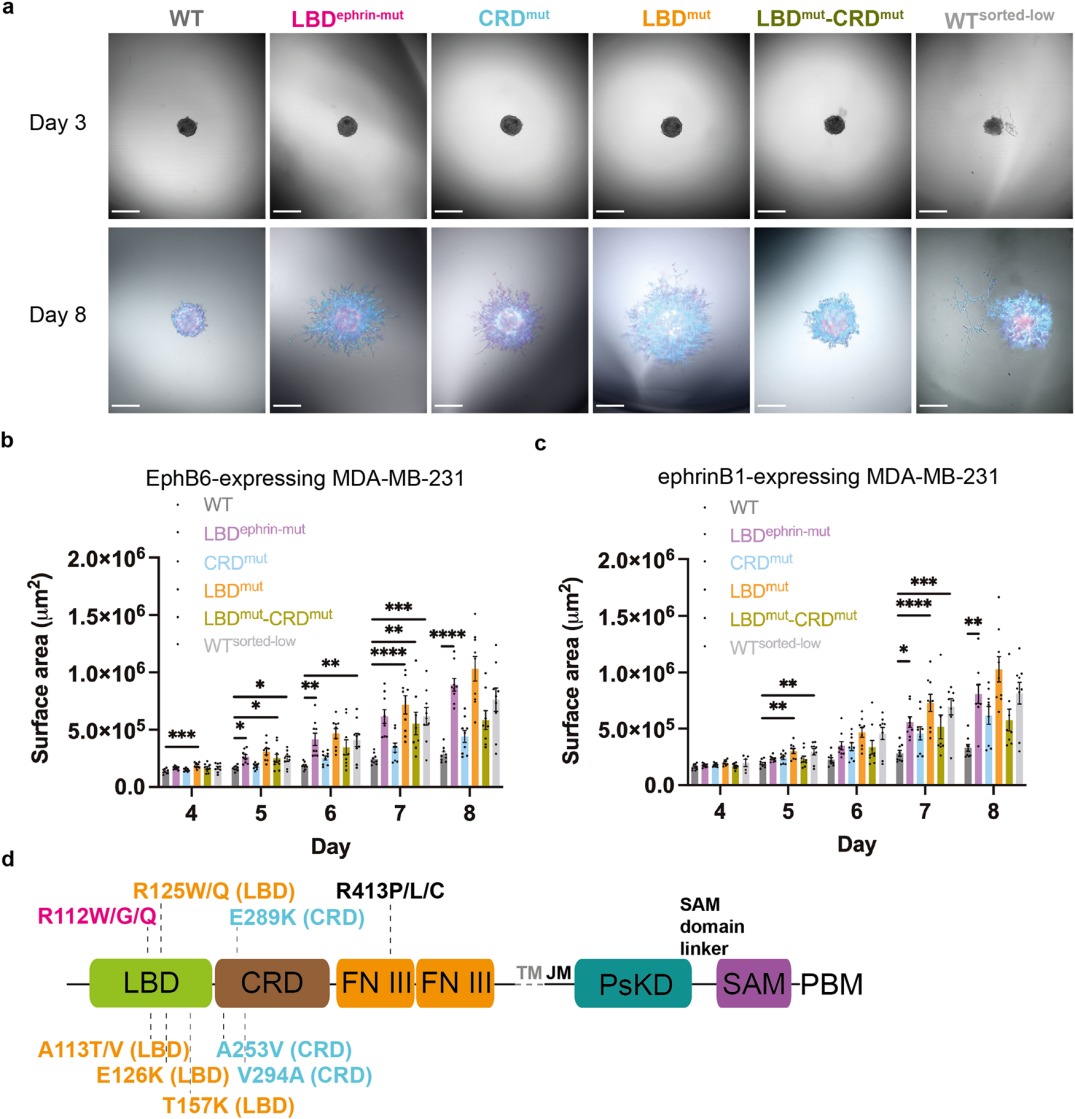
Co-clustering of EphB6 and ephrinB1 restrains cancer cell invasion. **a** Co-cultured EphB6-mNG-expressing MDA-MB-231 cells and ephrinB1-Halo-expressing MDA-MB-231 cells were grown as three-dimensional tumor spheroids. Invasion of MDA-MB-231 cells was monitored with the induction of EphB6-mNG and ephrinB1-Halo expression and the addition of the invasion matrix on Day 3. The scale bar indicates 500 µm. **b**, **c** The invasiveness of the MDA-MB-231 cells was evaluated by quantifying the area occupied by **b** the EphB6-mNG-expressing MDA-MB-231 cells, and **c** the ephrinB1-Halo-expressing MDA-MB-231 cells from various co-culture systems, by comparing their fluorescence emission colors (green fluorescence from the EphB6-mNG-expressing MDA-MB-231 cells and red fluorescence from the ephrinB1-Halo-expressing MDA-MB-231 cells, as the spheroids were grown in the presence of 50 nM JF549). *N* = total eight to nine spheroids from each co-culture system, from two biologically independent experiments. The data are represented by mean ± SEM (standard error of the mean). *P < 0.05, **P < 0.01, ***P < 0.001, and ****P < 0.0001 were calculated by one-way ANOVA followed by a Dunnett’s multiple comparison test. **d** EphB6 somatic mutations on the ligand-binding interface (colored in magenta), the CRD oligomerization interface (light blue), and the LBD oligomerization interface (orange) associated with solid tumors are mapped. A frequent mutation at the R413 on the N-terminal fibronectin III domain (FN III) is also included. Data source was from the Catalogue of Somatic Mutations in Cancer (COSMIC) database^44^.

We hypothesized that EphB6 might serve as an anchor for migrating cells through its interaction with an ephrin expressed by an adjacent cell and the subsequent formation of tubular structures. We speculated that these tubular structures could potentially suppress the invasiveness of cancer cells, considering the downregulation of EphB6 expression in metastatic cancer samples^14,16–18^, and the lack of detectable EphB6 in MDA-MB-231 cells (Supplementary Fig. 1f). Thus, to investigate the impact of the EphB6:ephrinB1 co-clusters on MDA-MB-231 cell invasiveness, we performed three-dimensional tumor spheroid invasion assays, monitoring the area of invasion on a focal plane. Initially, we examined the invasiveness of the MDA-MB-231 cells harboring exogenous EphB6 constructs, but not co-cultured with ephrinB1-expressing cells. Interestingly, we observed reduced invasiveness overall (Supplementary Fig. 6), consistent with previous findings for WT-EphB6^17^. To examine the potential suppressive effect of co-clustering with ephrinB1 on invasiveness, we co-cultured cells into tumor spheroids. We found the invading area of the spheroids containing the WT-EphB6-mNG and ephrinB1-Halo cell lines was two- to three-fold smaller compared to spheroids with each of the EphB6 mutants that exhibited reduced or abrogated capacity to form clusters (Fig. 5a). Time-lapse quantification of WT-EphB6-mNG-expressing cells also revealed that they were significantly less invasive (Fig. 5b). The repression of invasiveness primarily resulted from EphB6:ephrinB1 interaction, as the area occupied by the co-cultured ephrinB1-Halo-expressing MDA-MB-231 cells displayed the same trend of suppression (Fig. 5c). Notably, the cells with lower expression of WT-EphB6-mNG, termed WT-EphB6-mNG^sorted-low^, exhibited a similar amplitude of invasiveness to those of other EphB6 mutants (Fig. 5a–c), indicating that the EphB6 expression level may be important for curbing tumor cell invasion. Importantly, according to the Catalogue of Somatic Mutations in Cancer (COSMIC) database^44^, cancer-associated mutations are prevalent on the ligand-binding interface, the LBD and the CRD oligomerization interfaces of EphB6, including R112W/G/Q, E126K and V294A (Fig. 5d and Supplementary Fig. 1d). Our data demonstrated that perturbations of these residues abrogate ephrin binding and receptor oligomerization (Fig. 1e–h), in keeping with predictions of reduced co-clustering and cancer cell invasion (Fig. 5a–c). This supports the idea that perturbation of EphB6 clustering could serve as an underlying mechanism that drives cancer invasion.

## Discussion

The dysregulated phosphorylation resulting from overexpression and hyperactivity of RTKs can predisposes cells to malignant transformation^45,46^. Some RTKs, however, do not have kinase activity and thus are categorized as receptor tyrosine pseudokinases^8^. Intriguingly, these receptor tyrosine pseudokinases, including EphB6, have also been implicated in cancers^8,47^, indicating that their non-catalytic functions are biologically significant. In this study, we investigated the non-catalytic functions of EphB6 in a cellular context, revealing that upon engagement with a cognate ligand, ephrinB1, EphB6:ephrinB1 clustering occurs at the plasma membrane. Such interaction leads to the engulfment of membrane protrusions, followed by the formation of double-membrane tubular structures between interconnecting cells. To the best of our knowledge, such structures have not been reported previously. Furthermore, the strong association between EphB6 and ephrinB1 leads to a reduction in the invasiveness of the breast cancer cell line, MDA-MB-231, revealing how EphB6 might function as a tumor suppressor when interacting with ephrinB1 in trans.

The observation of the endocytosis-resistant EphB6:ephrinB1 co-clusters and tubular structures were unexpected findings that have not been reported in the MDA-MB-231 cellular model. In an analogous model, co-culture of EphA2- and ephrinA1-expressing MDA-MB-231 cells led to EphA2:ephrinA1 clustering at cell–cell junctions, but these clusters underwent endocytosis and did not give rise to tubular structures between cells^35^. Similarly, in co-culture experiments with EphB2- and ephrinB1-expressing HeLa cells, these clusters also underwent endocytosis and did not give rise to tubular structures^34^. While we note that the reliance on protein overexpression to study the cellular functions of Eph receptors is a limitation of our studies and those reported previously, we are able to make comparisons between our observations herein and earlier work. Most notably, ours studies reveal the unique characteristics of the EphB6:ephrinB1 co-clusters and tubular structures, which were not observed in earlier studies of catalytically active Eph receptors.

The receptor clustering propensity, the protein expression level, and the endocytosis rate of the clusters together determine the size of the Eph:ephrin co-clusters, and thus the likelihood of membrane protrusion engulfment. We showed that two oligomerization interfaces in the ectodomain’s of EphB6 govern receptor clustering; these interfaces are conserved amongst the Eph receptors, likely indicating a common mechanism for receptor clustering in the Eph receptor family. Additionally, our findings on the EphB1 receptor highlight a role for the catalytic activity of the kinase domain in promoting cluster endocytosis. However, even when EphB1’s kinase activity was impaired, its endocytosis rate was only halved and remains significantly higher than that of EphB6. This suggests that clustered EphB6 may possess unique properties that confer resistance to endocytosis. Consequently, resistance to endocytosis and persistence of these clusters on the plasma membrane may predispose EphB6-expressing cells to the formation of tubular structures when exposed to ephrinB1-expressing cells.

Tubular structures interconnecting cells are common. In 2004, a type of cell–cell communication involving tunneling nanotubes (TNTs) was first described^48^, although a precise definition of TNTs has been under debate since its discovery. Generally, TNTs are recognized as thin tubes that establish open-ended connections between two cells, allowing for the transport of various materials, including small molecules; macromolecules, such as proteins; and even organelles, such as mitochondria^49,50^. The mechanisms underlying the formation of TNTs remain unclear, but some theories propose their origin from lamellipodium- or filopodium-like membrane protrusions at the leading edge of the cells^51–53^. Once a membrane protrusion reaches another cell, fusion can occur resulting in the formation of an interconnected TNT^50,54^. In cultured cells, TNT are typically positioned over the Petri dishes^49^. In our study, most of the tubular structures decorated by the EphB6:ephrinB1 co-clusters are also hovering over. This hindered us from applying three-dimensional stochastic optical reconstruction microscopy (3D-STORM) in a total internal reflection fluorescence (TIRF) mode to characterize their morphology, as the reconstructed images had low signal-to-noise. However, these tubules are distinct from TNTs, because their formation relies on the interaction of EphB6 and ephrinB1 in trans and EphB6:ephrinB1 co-clustering. This implies that these tubular structures could be a different form of intercellular interaction that has not been previously described. We showed that the tubular structures arising from EphB6:ephrinB1 co-clustering possess unique morphological characteristics and are likely formed through the engulfment of membrane protrusions by using super-resolution microscopy, CLEM, and cryo-ET. Owing to the resolution limit of our cryo-ET workflow, direct visualization of the EphB6:ephrinB1 clusters on the double membranes was unsuccessful. While other mechanisms that lead to the formation of such tubular structures may exist, our proposed engulfment model was supported by the double-membrane morphology observed in our 3D SIM data and the tomogram. Interestingly, our live cell imaging demonstrated that the double-membrane structure of these structures enables the lateral diffusion of the EphB6:ephrinB1 clusters along the tubes, which would not be possible if these tubular structures consisted of a single membrane, as is typical of TNTs. While our study focuses on EphB6, it remains to be determined to what extent this mode of cell–cell interaction is employed by other receptors within the Eph receptor family. Nevertheless, our findings provide a foundation for understanding the evolutionary adaptation of catalytic inactivity in mammalian Eph receptor orthologs. Whether a similar mode in intercellular interaction occurs in fish, reptiles, and birds, where EphB6 contains residues typical of an active kinase domain^55^, also remains to be established. Overall, our findings contribute to the body of evidence supporting the notion that pseudoenzymes, in the absence of selective pressures to retain ancestral catalytic activities, can evolve distinct functions.

## Methods

### Cell lines

HEK293, HEK293T, and MDA-MB-231 cells were cultured in DMEM media supplemented with 7.5% fetal calf serum (FCS) and Penicillin (100–120 units/mL) and Streptomycin (100–120 µg/mL). Cells were maintained in a humidified incubator at 37 °C with 5% CO_2_ supply.

### DNA constructs for mammalian cell expression

All the human full-length, mNeonGreen tagged EphB6 sequences were synthesized and sequenced by Genscript. The human full-length EphB1 sequences were synthesized by Genewiz, and subcloned to generate WT-EphB1-mNG and D744N-EphB1-mNG. Their sequences were confirmed by Sanger sequencing (Micromon, Monash University, Australia) to ensure sequence authenticity. The human full-length, HaloTag conjugated ephrinB1 sequence was synthesized and sequenced by Genewiz. For EphB6 or EphB1, their complete DNA constructs encode the EphB6 or EphB1 signal peptide sequence (residues 1–31 for EphB6, and residues 1–17 for EphB1, predicted by SignalP 3.0^56^ and SignalP 5.0^57^, respectively), a Gly-Ser linker, a Flag tag sequence (DYKDDDDK), another Gly-Ser linker (resulting from a BamHI cloning site), the EphB6 (residues 32–1015) or the EphB1 (residues 18–978) sequences, a (Gly-Gly-Ser)_5_ linker, the mNeonGreen sequence, followed by the EphB6 (residues 1016–1021) or EphB1 (residues 979- 984) PDZ domain-binding motif. To generate human full-length ephrinB1, the same design strategy was used. The ephrinB1 signal peptide sequence (residues 1–27, predicted by SignalP 3.0^56^) was followed by an HA tag (YPYDVPDYA), the ephrinB1 (residues 28–340) sequence, a (Gly-Gly-Ser)_5_ linker, the HaloTag sequence and the C-terminal PDZ domain-binding motif (residues 341–346). All the Eph receptors- and ephrinB1-related DNA constructs were subcloned into a destination vector, pFTRE3G, which is a puromycin-selective, and doxycycline-inducible lentiviral vector, a generous gift from Dr Toru Okamoto^58^. The cloning sites of the inserts were BamHI and EcoRI, whereas those of the pFTRE3G vector were BglII and EcoRI, resulting in a non-cleavable scar sequence (GGATCT).

### Generation of the EphB6, EphB1 and ephrinB1 doxycycline-inducible expression system in MDA-MB-231 cells

A lentiviral system was used to generate stable cell lines expressing the aforementioned EphB6, EphB1, and ephrinB1 constructs upon doxycycline induction. Transfection was performed by incubating HEK293T cells with pFTRE3G vector encoding EphB6, EphB1 or ephrinB1, with pCMV ΔR8.2 and pVSVg helper plasmids, using the Effectene Transfection Kit (Qiagen, Germany). First, HEK293T cells were seeded in a 10 cm Petri dish to reach ~80% confluency on the day of transfection. Each transfection started by mixing 2 μg of pFTRE3G vector encoding EphB6, EphB1, or ephrinB1 variants with 1 μg of pCMV ΔR8.2 and 1 μg of pVSVg in 802 μL of EC Buffer provided in the Transfection Kit (Qiagen, Germany). After incubation for 10 s, 32 μL of Enhancer was added to each reaction mixture, and incubated for 5 min at room temperature. 32 μL of Effectene was then added and the reaction mixture was further incubated for 10 min at room temperature before being added to the HEK293T cells in a dropwise manner. The transfected cells were incubated for 48 h in a humidified incubator at 37 °C with 5% CO_2_ supply. Meanwhile, MDA-MB-231 cells were seeded into 6-well cell culture plates, which are to be infected by the lentivirus. The resulting supernatant from the transfected HEK293T cells after 48 h became the lentivirus, which was then harvested and filtered through a 0.45 μm filter. Polybrene, a polymer that can enhance the transfection efficiency, was added to the lentivirus a final concentration of 8 µg/mL. When the pre-seeded MDA-MB-231 cells reached ~60% confluency, the media were replaced with lentivirus and spin infection was performed at 750 × *g*, 30 °C for 45 min. After another 48 h incubation, puromycin selection was carried out on the MDA-MB-231 cells at a final concentration of 2.5 µg/mL. The puromycin selection process of the lentivirus-infected MDA-MB-231 cells proceeded for 1 week, which led to the MDA-MB-231 stable cell lines capable of expressing EphB6, EphB1 or ephrinB1 variants upon induction by 20 ng/mL doxycycline.

### Western blotting

Cell lysates or immunoprecipitated proteins were denatured and reduced in 4× Reducing Sample Buffer (40% Glycerol, 240 mM Tris-Cl, 8% SDS, 0.4 M DTT, and 0.1% Bromophenol blue, pH = 6.8), heated at 100 °C for 5 min. The samples were then loaded on a NuPAGE 4–12%, Bis-Tris, 1.0 mm, Mini Protein Gel (ThermoFisher, US), and run in a tank filled with NuPAGE MES SDS Running Buffer at 150 mV for 60 min at room temperature. The resolved proteins were transferred to PVDF membrane (Transfer membrane Immobilon-P PVDF, Millipore, US) at 90 mV at 4 °C for 1.5 h in Transfer Buffer (25 mM Tris, 192 mM Glycine, 20% Methanol, and 0.1% SDS in water, pH = 8.3), followed by blocking the PVDF membrane in 5% skim milk in PBS (with 0.1% Tween 20) at room temperature for 30 min. The PVDF membrane was then rinsed with PBST for 5 min three times and was probed with the corresponding primary antibody in 5% skim milk or bovine serum albumin (BSA) solution in PBST at 4 °C overnight. The next day, primary antibody probed membrane was washed with PBST for 5 min three times prior to incubating with the HRP-conjugated secondary antibodies at room temperature for 1 h. After rinsing with PBST for 5 min three times, the membrane was developed in Immobilon Western Chemiluminescent HRP Substrate (Cat# WBKLS0500, Merck, US), and images of the blots were taken using a ChemiDoc (Biorad, US).

### Live cell imaging – confocal microscopy

The day before doxycycline induction, 6 × 10^4^ EphB6, EphB1, and/or ephrinB1-expressing MDA-MB-231 cells/well were seeded on a µ-Slide 8-well chamber coverslip (ibidi, Germany). Protein expression was induced by 20 ng/mL doxycycline for 16–24 h. Depending on the purposes of imaging, dyes with the corresponding concentration listed in Supplementary Table 1 were added to the cell culture and incubated at 37 °C for 1 h prior to imaging. The cell culture media were then replaced with Leibovitz’s L-15 media (GIBCO, ThermoFisher Cat# 21083027) (+7.5% FCS), to reduce background fluorescence from the dyes and the Phenol Red in the DMEM media. The confocal images were obtained by a Zeiss LSM980 Fast Airyscan 2 confocal microscope. A humidity chamber with temperature set at 37 °C was used during the experiments. The microscope is equipped with an Argon laser with the wavelength of 488 nm, a diode laser with the wavelength of 561 nm, and a HeNe laser with the wavelength of 639 nm. Objectives with a 40× magnification and a 1.3 numerical aperture (NA), or with a 63× magnification and a 1.4 NA were used. The objectives were immersed in Carl Zeiss Immersol Immersion Oil 518F (Germany) with a refractive index of 1.518 at 37 °C. A 32-channel GaAsP array detector was used to acquire time-lapsed images or Z-stack scanning. The images acquired were presented as maximum-intensity projections in Fiji. The contrast of the images was adjusted to enhance the signal-to-noise ratio and for optimal visualization.

For quantitation of clustering of different EphB6 variants, 50 nM JF646 (100 µM stock solution in DMSO) was used to label ephrinB1-Halo, and confocal images were taken. Each field of view was counted as a data point. From the co-culture systems with different EphB6 variants, all the tubular structures interconnecting an EphB6- and an ephrinB1-expressing cell were manually selected in a blinded manner in Fiji. After that, quantitation of the fluorescence intensities of EphB6 and ephrinB1 on these tubules was automated in Fiji. This allows determination of the tubules with co-clusters, which is required to fulfill a stringent threshold: 0.9 < the ratio of the fluorescence intensities of EphB6 and ephrinB1 < 1.1. The final “% clustering” of each EphB6 variant presented in Fig. 1i = the number of tubules with co-clusters/the number of total tubules.

### Live cell imaging – lattice light-sheet microscopy (LLSM)

Co-culture of ephrinB1-expressing HEK293 cells^59^ and EphB6-expressing MDA-MB-231 cells was imaged by the LLSM^60^ housed in the Centre for Dynamic Imaging at the Walter and Eliza Hall Institute of Medical Research. The plasma membrane of the ephrinB1-expressing HEK293 cells was visualized by transfecting the cells with mTagRFP-Membrane-1 (Plasmid #57992, Addgene) using the same protocol for generating the MDA-MB-231 stable cell lines. Co-cultured cells were grown on 5 mm round glass coverslips (Warner Instruments, Cat# CS-5R). During imaging, cells were maintained in Leibovitz’s L-15 media supplemented with 7.5% FBS. Samples were illuminated by lattice light-sheet using 488 nm and 560 nm diode lasers (MPB Communications) through an excitation objective (Special Optics, 0.65 NA, 3.74-mm WD). The lattice light-sheet was illuminated to the back aperture of the excitation objective through an annular mask of 0.44 inner NA and 0.55 outer NA. Fluorescent emission was collected by detection objective (Nikon, CFI Apo LWD 25XW, 1.1 NA), and detected by an sCMOS camera (Hamamatsu Orca Flash 4.0 v2). Acquired data were deskewed as previously described^60^ and deconvolved using an iterative Richardson-Lucy algorithm. Point-spread functions for deconvolution were experimentally measured using 200 nm TetraSpeck beads adhered to 5 mm glass coverslips (Invitrogen, Cat# T7280) for each excitation wavelength. Data is presented and visualized as maximum-intensity projections. All the other lattice light-sheet microscopy experiments were performed on a Lattice Lightsheet 7 microscope (Zeiss – Pre-serial). The microscope is equipped with diode lasers with wavelengths of 488, 561, and 640 nm, respectively. An excitation objective with a 13.3x magnification and a 0.44 numerical aperture (NA) was used. A detection objective with a magnification of 44.93× magnification and a 1 NA was applied. A 30 µm × 1 µm light-sheet was used for all image acquisition. The image interval along the Y-axis was set at 0.3 µm with a total range of 250 µm. The laser power was set between 5–10% with an exposure time of 5–10 ms based on the fluorescence intensities of different fluorophores used in the experiments. Aberrations were corrected using an aberration correction value of 182. Auto-immersion was used every 13 min. A humidity chamber with temperature control and 10% CO_2_ supply was used during live cell imaging. The acquired images were deskewed and deconvolved using ZEN (Zeiss), and the post-processed images were presented as maximum-intensity projections in Fiji. The contrast of the images was adjusted to enhance the signal-to-noise ratio and for optimal visualization.

For quantitation of the endocytosis rate of the EphB6:ephrinB1 and EphB1:ephrinB1 co-clusters, 50 nM JFX650 (100 µM stock solution in DMSO) was used to label ephrinB1-Halo and live cell imaging by LLSM with a duration of 30 min was taken for each experimental repeat. The sites at which the membrane protrusion of the ephrinB1-Halo-expressing cells was in contact with the EphB6-mNG- or EphB1-expressing cells, and at which the co-cluster was visible, was selected for quantitation. The number of the endocytosis events was counted manually in a blinded manner. The resulting quantitative analysis was plotted in Prism. For quantitation of the formation of tubules decorated by the EphB6:ephrinB1 co-clusters, 50 nM JF646 or JFX650 (100 µM stock solution in DMSO) was used to label ephrinB1-Halo and live cell imaging by LLSM with a duration of 2–3 h was conducted for each experimental repeat. The newly established tubules between EphB6- and ephrinB1-expressing cells during image acquisitions were manually selected and quantified based on whether they originated from the receptor- or ligand-expressing cells.

### Fixation of cells

6 × 10^4^ WT-EphB6-mNG-expressing and ephrinB1-Halo expressing MDA-MB-231 cells / well were seeded onto a µ-Slide 8-well chambered coverslip with glass bottom (ibidi, Germany). The co-cultured cells were induced by 20 ng/mL doxycycline for 16–24 h prior to chemical fixation. The fixation was performed by first rinsing the cells with PBS three times, followed by incubating with 0.05% glutaraldehyde + 2% paraformaldehyde in PBS at room temperature for 15 min and 4% paraformaldehyde in PBS for another 15 min^61^. The fixed cells were rinsed with PBS for three times to remove the crosslinkers and stored in PBS at 4 °C prior to the subsequent experimental procedure.

### Three-dimensional structured illumination microscopy (3D SIM) experiments

Super-resolution three-dimensional structured illumination microscopy (3D SIM) was performed on the DeltaVision OMX-SR system (GE Healthcare) equipped with a 60×/1.42 N.A. PlanApo oil immersion objective (Olympus), sCMOS cameras, and 488 and 568 nm lasers, and 1.516 refractive index immersion oil. 3D SIM image stacks were acquired consisting of 15 raw images per plane (5 phases, 3 angles) per color channel and a z-step size of 125 nm. Super-resolution reconstruction and color channel alignment were performed with softWoRx 7.0 (GE Healthcare).

### Fluorescence-activated cell sorting (FACS)

To obtain a population of MDA-MB-231 cells with a lower expression level of WT-EphB6-mNG, and a higher expression level of WT-EphB1-mNG and D744N-EphB1-mNG. Two runs of flow cytometry experiments were performed by a BD FACSAria™ III Cell Sorter (Biosciences) using a 100 µm nozzle. For the first run of sorting: WT-EphB6-mNG-expressing cells were pre-induced by 20 ng/mL doxycycline for 24 h, followed by trypsinization and sorting to collect a 9.6% cell population with the lowest WT-EphB6-mNG expression level; WT-EphB1-mNG and D744N-EphB1-mNG-expressing cells were pre-induced by 100 ng/mL doxycycline for 24 h, followed by trypsinization and sorting to collect a 7.6% and 2.4% cell population with the highest WT-EphB1-mNG and D744N-EphB1-mNG expression level, respectively. After expanding the sorted cells, they were subjected to the second run of sorting: WT-EphB6-mNG-expressing cells were pre-induced by 20 ng/mL doxycycline for 24 h, followed by trypsinization and sorting to collect a 4.8% cell population with the lowest WT-EphB6-mNG expression level; WT-EphB1-mNG and D744N-EphB1-mNG-expressing cells were pre-induced by 20 ng/mL doxycycline for 24 h, followed by trypsinization and sorting to collect a 25.5% and 24.6% cell population with the highest WT-EphB1-mNG and D744N-EphB1-mNG expression level, respectively. This resulted in the final population of cells, termed WT-EphB6-mNG^sorted-low^, WT-EphB1-mNG^sorted-high^, D744N-EphB1-mNG^sorted-high^, that were used to determine the endocytosis rate, when co-clustering with ephrinB1.

### Seeding co-cultured cells on EM grids

Quantifoil R 2/2 200 Mesh, extra thick, Gold (Electron Microscopy Sciences, US) EM grids were used for all the cryo-electron tomography work. An EM grid placed in a 35 mm dish (µ-Dish 35 mm, low, polymer bottom) (ibidi, Germany) was soaked in PBS overnight. On the next day, PBS was removed, and trypsinized cells were carefully seeded to each 35 mm dish, with 6 × 10^5^ WT-EphB6-mNG-expressing MDA-MB-231 cells and 6 × 10^5^ ephrinB1-Halo-expressing MDA-MB-231 cells per 35 mm dish. The next day, WT-EphB6-mNG and ephrinB1-Halo expression was induced by 20 ng/mL doxycycline for 16–18 h. Prior to chemically fixing and plunge-freezing the cells on the grids, JF549 (100 µM stock solution in DMSO) with a final concentration of 50 nM was added to the cells for a 1 h at 37 °C, to allow identification of the EphB6:ephrinB1 clusters in the subsequent CLEM workflow.

### Preparation of concentrated 10 nm gold colloidal solution for co-cultured cells on EM grids

Colloidal gold solution was used to deposit fiducial markers for the cryo-electron tomography. 1 mL of unconjugated 10 nm colloidal gold solution (Ted Pella, US) was mixed with 250 µL of 5% BSA solution in PBS. After gentle mixing, the suspension was centrifuged at 20000 × *g* for 30 min at 4 °C. 1.2 mL of the supernatant was removed to leave the concentrated colloidal gold solution.

### Plunge-freezing the EM grids

A manual, thin-film freezing apparatus in a humidity-controlled room (20% relative humidity) was used to plunge freeze the EM grids. The manual plunger was pre-cooled by liquid nitrogen, and the sample chamber in which the EM grids are to be frozen was then filled with liquid ethane. When the ethane had just begun to solidify, an EM grid with co-cultured cells was retrieved using a Dumont L5 tweezer, and mounted on the manual plunger. 4 µL of the concentrated 10 nm colloidal gold solution was applied onto the side of the EM grids on which cells were seeded. A piece of filter paper (Whatman #1, GE) was used to blot the EM grids from the back side for 12 s to avoid any damage to the cells, followed by immediate plunge-freezing in liquid ethane cooled by liquid nitrogen. The grids were stored in liquid nitrogen prior to cryo-electron tomography experiments.

### Correlative light and electron microscopy (CLEM) workflow

Cells fixed on the EM grids were imaged by a Zeiss Axio Observer Widefield microscope. The microscope is equipped with LED laser sources with wavelengths of 470 nm and 555 nm. A water objective with 40× magnification and 1.2 numerical aperture was used. The objective was immersed in Carl Zeiss Immersol Immersion Water (Germany) with a refractive index of 1.334 at 23 °C. A tile scan of the whole EM grids was performed, recording the green and red fluorescence, and the brightfield channels by a sCMOS 16-bit camera. The individual tile scan images were stitched and presented in Fiji, which served as a light microscopy atlas.

The same EM grid with a light microscopy atlas generated was then subjected to the plunge-freezing protocol. The AutoGrids were loaded to a Titan Krios G4 cryo-transmission electron microscope. An electron microscopy atlas was derived by imaging the EM grid with a 135× magnification. The light and electron microscopy atlases were correlated, and the tubular structures associated with EphB6:ephrinB1 clusters were identified for the subsequent cryo-electron tomography experiments.

### Cryo-electron tomography (cryo-ET) experiments

Cryo-ET was performed on a Titan Krios G4 cryo-transmission electron microscope (cryo-TEM) (ThermoFisher, US) operated at 300 keV. A Bioquantum K3 (Gatan, US) direct electron detector with pixel dimensions of 5.8 K × 4 K was employed for cryo-ET experiments. This is because it has a 1.5× larger field of view and it can be conjugated with a Gatan imaging filter (BioContinuum GIF, Gatan, US), and operated in a zero-loss mode to remove inelastically scattered electrons that cause image blur. Prior to performing cryo-ET, optical path alignment and correction of the cryo-TEM was performed to ensure optimal image acquisition. This included direct column alignments (gun tilt, gun shift, pivot points, condenser lenses, and rotation center). Scherzer focus was calibrated using the Fast Fourier Transform (FFT) of live micrographs so that autofocusing on an adjacent region along the tilt axis produced micrographs with the desired defocus, i.e. −6 µm. Images were acquired using the SerialEM software. Tilt series were collected over an angular range of –67.5° to +67.5° with a 1.5° increment using the dose symmetric (Hagen) scheme^62,63^ to aid in automating serial collection of tomograms, with a total accumulative dose of 2700–3600 e/nm^2^ per tilt series. The tilt series were recorded using a nominal 26000x magnification, corresponding to a pixel size of 3.4 Å. Tilt series were processed, and low-quality frames were removed using IMOD (version 4.11)^64^. The tilt series was binned by a factor of 2 to a pixel size of 6.8 Å/pixel. The tilt series was manually aligned using Etomo interactive (an IMOD plugin in Scipion). Fiducials were used for tracking and the final loss was below <0.5; however, the fiducials were poorly distributed so even though the tracking of the beads was satisfactory, the “global” tracking was suboptimal. The aligned tilt series was binned to 13.56 Å/pixel and used for final reconstruction. An 800 voxel thick reconstruction was done using tomo3d simultaneous iterative reconstruction (SIRT) (30 iterations) as a plugin in Scipion^65^. To increase contrast, the tomogram was deconvoluted using IsoNet with the following parameters: pixel size 13.56 Å, defocus 5.7 µm, snrfalloff 1.0, and deconvstrength 1.0. The reconstructed three-dimensional volumes were viewed in an interpolated mode.

### AlphaFold2 structure prediction

The ColabFold Google Colab notebook AlphaFold2^66,67^ was used to predict the structure of full-length EphB6 (Entry: O15197) and ephrinB1 (Entry: P98172). The predicted models with the highest lDDT score were used to conduct our structural analysis.

### Spheroid invasion assays – spheroid formation and invasion

On day 0, 1 × 10^3^ EphB6-mNG MDA-MB-231 cells and 1 × 10^3^ ephrinB1-Halo MDA-MB-231 cells, with 1× Cultrex Spheroid Formation Extracellular Matrix (R&D, Cat# 3500-096-01) were mixed to a volume of 60 µL per well, and added to a 96-well Nunclon Sphera-Treated, U-Shaped-Bottom Microplate (ThermoFisher Scientific, Cat# 174925). The plate was spun at 200 × *g* for 3 min at room temperature, followed by incubation at 37 °C for 72 h. On day 3, 50 µL Cultrex Spheroid Invasion Extracellular Matrix (R&D, Cat# 3500-096-03) per well were added to the 96-well microplate, which has been pre-cooled on ice for 20 min. The plate was then spun at 300 × *g* for 3 min at 4 °C, followed by incubation at 37 °C for 40 min. 100 µL of pre-warmed Complete DMEM media (DMEM media with 10% FBS, 40 ng/mL doxycycline, and 50 nM JF549) per well were added to induce protein expression and allow visualization of invasion of the cells in the subsequent imaging experiments. On day 6, 30 µL per well of the old media were replaced with 60 µL fresh Complete DMEM media.

### Spheroid invasion assays – image acquisition and quantification

From Day 3, an image of each well for a 96-well Nunclon Sphera-Treated, U-Shaped-Bottom Microplate, was taken every 24 h, using a widefield microscope (Zeiss Axio Observer). The microscope was equipped with a chamber set at 37 °C and supplied with 5% CO_2_. A 5×/NA = 0.16 objective was used with air immersion. LEDs at 470 nm and 555 nm were used to excite EphB6-mNeonGreen and ephrinB1-Halo (with 50 nM JF549), respectively. Both channels had an LED power of 40% with an exposure time of 800 ms across all the wells. Transmitted light illumination was also applied to obtain brightfield images, at a power of 3–10%. Images were acquired at a focal plan to capture a best overview of the spheroids, where the largest surface area of the spheroids could be recorded. The resulting images were analyzed in Fiji. The contours of the spheroids were selected manually to estimate the size of the area occupied by the spheroids. For the co-culture systems, an identical threshold of intensities set manually for the green fluorescence was applied to quantify the area occupied by the EphB6-mNG-expressing cells in a focal plane, across all EphB6 variants, to best reflect the occupancy of the spheroids. Likewise, the same procedure was applied to quantify the area occupied by the ephrinB1-Halo-expressing by detecting the red fluorescence. The resulting quantitative analysis was plotted in Prism.

### Reporting summary

Further information on research design is available in the Nature Portfolio Reporting Summary linked to this article.

### Supplementary information

Supplementary Information

Description of Additional Supplementary Files

Supplementary Data

Supplementary Movie 1

Supplementary Movie 2

Supplementary Movie 3

Supplementary Movie 4

Supplementary Movie 5

Supplementary Movie 6

Supplementary Movie 7

Supplementary Movie 8

Reporting summary

## Data Availability

Numerical source data are available in the Supplementary Data file, unprocessed blots/gel images are available as Supplementary Fig. 7. All other data are available from the corresponding authors upon request.
