# Supplementary material for: Co-clustering of EphB6 and ephrinB1 in trans restrains cancer cell invasion
Source: Commun Biol. 2024 Apr 16;7:461. doi: 10.1038/s42003-024-06118-4 (PMC11021433; doi:10.1038/s42003-024-06118-4)
Supplement: Supplementary file 1 — Supplementary Information [file 42003_2024_6118_MOESM1_ESM.pdf]

Supplementary Information

Co-clustering of EphB6 and ephrinB1 *in trans* restrains cancer cell invasion

Lung-Yu Liang[1,2], Niall D. Geoghegan[1,2], Michael Mlodzianoski[1,2], Andrew Leis[1,2], Lachlan W. Whitehead[1,2], Minglyanna G. Surudoi[1,2], Samuel Young[1,2], Peter Janes[3], Doulin Shepherd[4], Debnath Ghosal[4,6], Kelly L. Rogers[1,2], James M Murphy[1,2,5*, #], Isabelle S Lucet[1,2,6*, #].

[1] Walter and Eliza Hall Institute or Medical Research, 1G Royal Parade, Parkville, VIC 3052, Australia
[2] Department of Medical Biology, University of Melbourne, 1G Royal Parade, Parkville, VIC 3052, Australia
[3] Olivia Newton-John Cancer Research Institute and La Trobe School of Cancer Medicine, Level 5, ONJ Centre, 145 Studley Rd, Heidelberg, VIC 3084, Australia
[4] Department of Biochemistry and Pharmacology, Bio21 Molecular Science and Biotechnology Institute, The University of Melbourne, Melbourne, VIC, Australia.
[5] Drug Discovery Biology, Monash Institute of Pharmaceutical Sciences, Monash University, Parkville, VIC 3052, Australia
[6] ARC Centre for Cryo-electron Microscopy of Membrane Proteins, Bio21 Molecular Science and Biotechnology Institute, University of Melbourne, Parkville, Victoria, Australia

[*] These authors jointly supervised this work
[#]Correspondence: jamesm@wehi.edu.au, lucet.i@wehi.edu.au,

LIST OF SUPP FIGURES and SUPP MOVIES

Supplementary Table 1: Dyes used in this study

Supplementary Fig. 1: Generation of EphB6-mNG-expressing and ephrinB1-Halo-expressing MDA-MB-231 cells

Supplementary Fig. 2: Generation of EphB1-mNG-expressing MDA-MB-231 cells

Supplementary Fig. 3: Characterization of the tubular structures decorated by the EphB6:ephrinB1 co-clusters, after chemical fixation of the co-cultured cells

Supplementary Fig. 4: The CLEM and cryo-ET workflow

Supplementary Fig. 5: A structural model of the EphB6:ephrinB1 ectodomain protein complex fits the spacing between the double membranes of the tubular structures decorated by the EphB6:ephrinB1 clusters

Supplementary Fig. 6: Evaluation of the EphB6 expression on the invasiveness of cancer cells

Supplementary Fig. 7: Uncropped and unedited blot/gel images

Supplementary Table 1

Name	Excitation/ Emission	Working concentration	Application
JF549- HaloTag ligand*	549 nm/ 571 nm	50 nM	Labelling of ephrinB1-HaloTag for both live and fixed cell imaging
JF646- HaloTag ligand*	646 nm/ 664 nm	50 nM	Labelling of ephrinB1-HaloTag for both live and fixed cell imaging
JFX650- HaloTag ligand*	650 nm/ 667 nm	50 nM	Labelling of ephrinB1-HaloTag for both live and fixed cell imaging
CellMask Orange (Cat#. C10045, ThermoFisher)	554 nm/ 567 nm	5 μg/mL	Labelling of the plasma membrane for both live and fixed cell imaging

*A generous gift from Dr Luke Lavis at the Janelia Research Campus

Supplementary Figures.

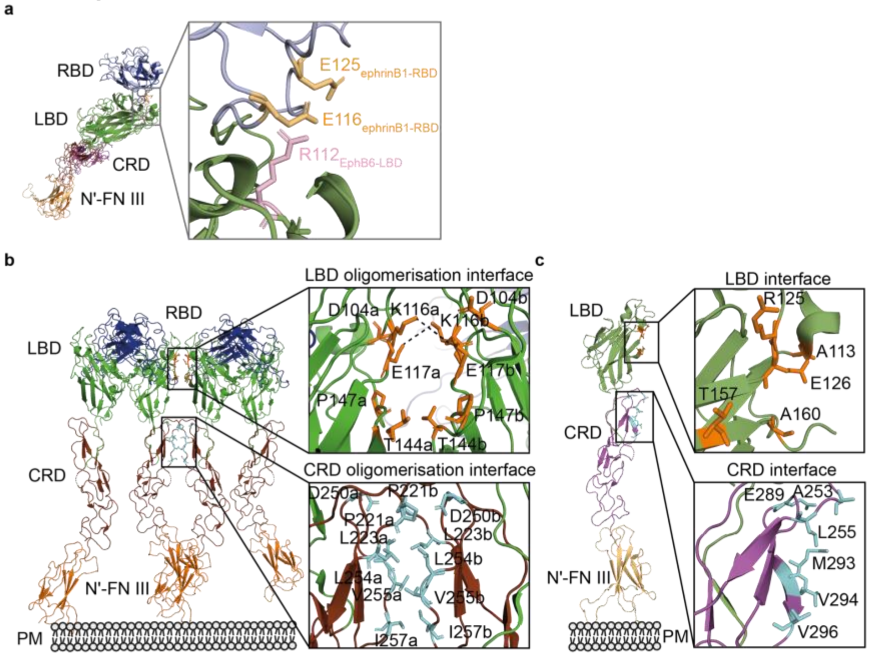

d

	LBD-LBD oligomerisation interface					CRD-CRD oligomerisation interface					
**EphB6**	A113	R125	**E126**	T157	A160	A253	**L255**	E289	**M293**	**V294**	V296
EphA2	D104	K116	E117	T144	P147	P221	L223	D250	L254	V255	I257
EphB1	D95	K107	E108	T139	A142	T216	L218	D243	M247	V248	I250

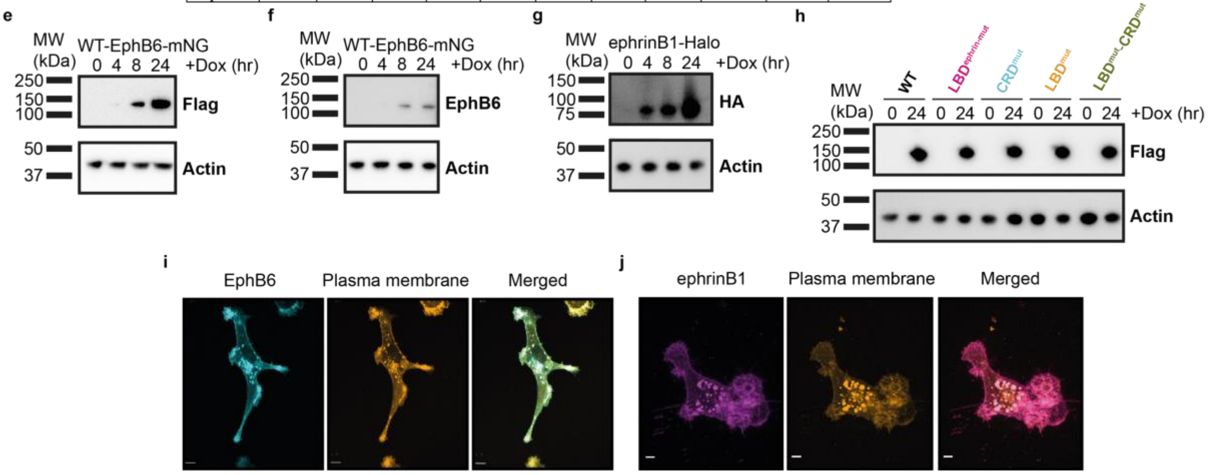

Supplementary Fig. 1: Generation of EphB6-mNG-expressing and ephrinB1-Halo-expressing MDA-MB-231 cells

a The crystal structure of the ectodomains of EphB6 (PDB: 7K7J [1]) and the RBD of ephrinB1 (PDB: 6THG [2]) were superimposed to the crystal structures of ephrinA5-liganded EphA2 (PDB: 3MX0 [3]), rendering a RMSD of 3.324 Å and 1.503 Å, respectively. E125 in the G-H loop of ephrinB1 is equivalent to the Glu residue used by other Eph receptor/ephrin pairs to form a hydrogen bond with the corresponding Arg residue (e.g. R112 of EphB6) in the Eph receptor LBD. **b** The residues comprising the LBD-LBD and CRD-CRD oligomerization interfaces of the EphA2:ephrinA5 ectodomain protein complex structure (PDB: 3MX0 [3]) are highlighted. RBD: receptor-binding domain. LBD: ligand-binding domain. CRD: cysteine-rich

domain. N'-FN III: N-terminal fibronectin domain III. PM: plasma membrane. **c** The residues comprising the putative LBD-LBD and CRD-CRD oligomerization interfaces of the EphB6 ectodomain structure (PDB: 7K7J [1]) are highlighted. The cartoons were made in PyMOL. **d** The residues comprising the LBD-LBD and CRD-CRD oligomerization interfaces of the EphA2:ephrinA5 ectodomain protein complex structure are listed along with the corresponding residues in EphB6. The equivalent residues in a Type B Eph receptor, EphB1, are also listed to exemplify that many of these residues are universally conserved across Type A and B Eph receptors. **e** Timecourse of WT-EphB6-mNG expression following induction with 20 ng/mL doxycycline, and examined by western blotting using an anti-Flag antibody. An anti-actin western blotting was performed as a loading control. Dox: doxycycline. **f** Timecourse of WT-EphB6-mNG expression following induction with 20 ng/mL doxycycline, and examined by western blotting using a commercial anti-EphB6 antibody. An anti-actin western blotting was performed as a loading control. **g** Timecourse of ephrinB1-Halo expression following induction with 20 ng/mL doxycycline, and examined by western blotting using an anti-HA antibody. An anti-actin western blotting was performed as a loading control. **h** Comparison of the expression level of WT-EphB6-mNG, LBD$^{ephrin\text{-}mut}$-EphB6-mNG, CRD^{mut}-EphB6-mNG, LBD^{mut}-EphB6-mNG and LBD^{mut}-CRD^{mut}-EphB6-mNG was examined by western blotting using an anti-Flag antibody. An anti-actin western blotting was performed as a loading control. Live cell imaging showing the membrane localisation of i WT-EphB6-mNG and j ephrinB1-Halo in the MDA-MB-231 cells. The plasma membrane was stained with CellMask Orange to confirm the localisation of the tagged proteins. The scale bar indicates 10 µm.

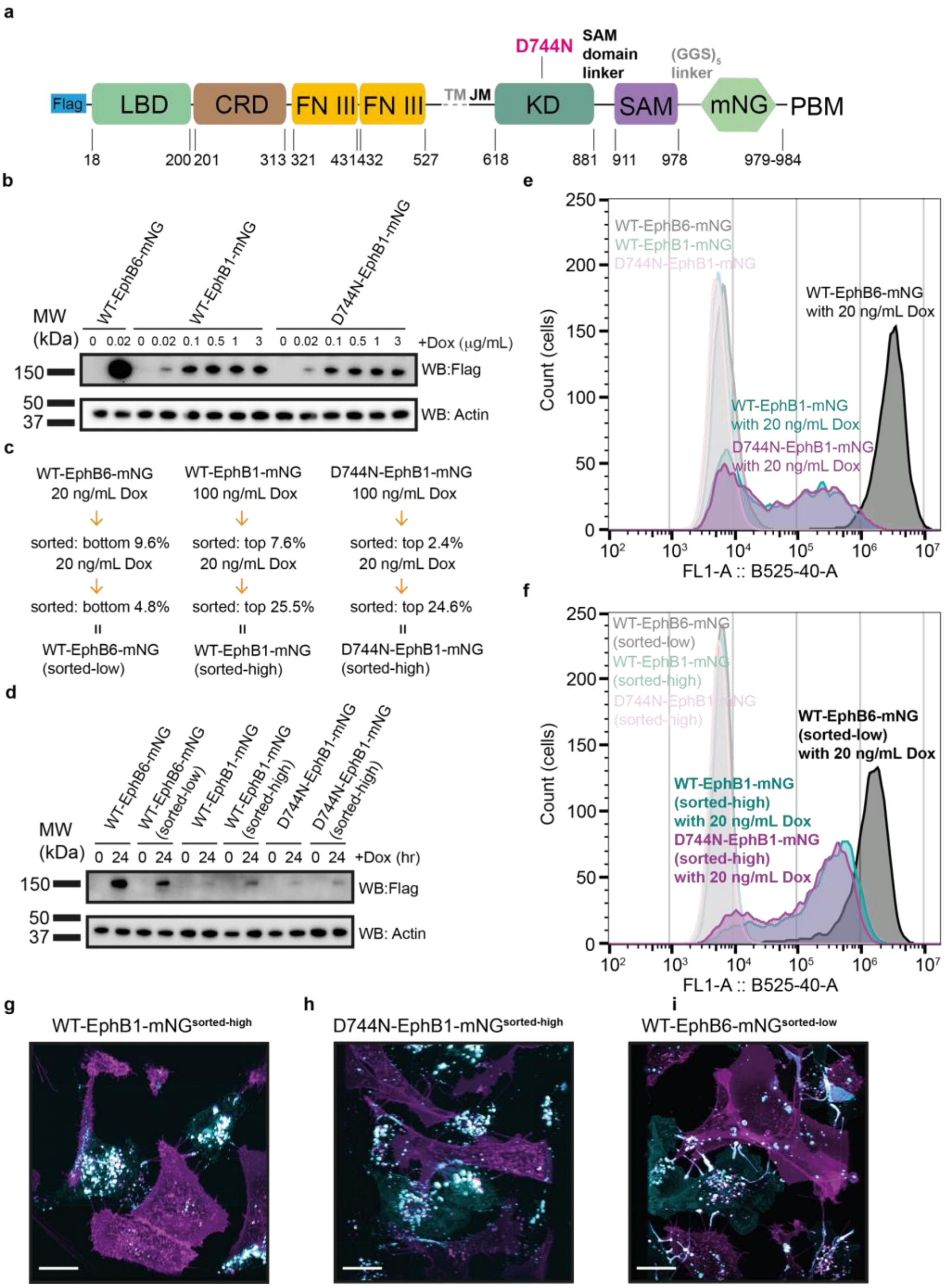

Supplementary Fig. 2: Generation of EphB1-mNG-expressing MDA-MB-231 cells

a The wild-type and mutant EphB1-mNG constructs. Flag: FLAG tag. LBD: ligand-binding domain. CRD: cysteine-rich domain. FN III: fibronectin III domain. TM: transmembrane domain. JM: juxtamembrane region. KD: kinase domain. SAM: sterile α-motif. mNG: mNeonGreen. PBM: PDZ domain-binding motif. **b** The doxycycline concentration-dependent expression of WT-EphB6-mNG, WT-EphB1-mNG and D744N-EphB1-mNG was examined by western blotting using an anti-Flag antibody after doxycycline induction for 24 hrs. An anti-actin western blotting was performed as a loading control. **c** The flow cytometry procedure to

sort out low expressers (indicated as “sorted-low”) of WT-EphB6-mNG-expressing MDA-MB-231 cells, and high expressers (indicated as “sorted-high”) of WT- and D744N-EphB1-mNG-expressing MDA-MB-231 cells. **d** Comparison of the expression level of WT-EphB6-mNG (original and sorted-low), WT-EphB1-mNG (original and sorted-high) and D744N-EphB1-mNG (original and sorted-high) was examined by western blotting using an anti-Flag antibody. An anti-actin western blotting was performed as a loading control. The flow cytometry histograms showing the green fluorescence intensities of **e** the original cell lines and **f** the ones after sorting by the flow cytometry, indicative of the protein expression level of EphB6-mNG and EphB1-mNG, in the presence and absence of 20 ng/mL doxycycline. Live cell imaging of the **g** WT-EphB1-mNG$^{\text{sorted-high}}$, **h** D744N-EphB1-mNG$^{\text{sorted-high}}$, and **i** WT-EphB6-mNG $^{\text{sorted-low}}$-expressing MDA-MB-231 cells (in cyan), co-cultured with the ephrinB1-Halo-expressing MDA-MB-231 cells (in magenta). The scale bar indicates 20 µm.

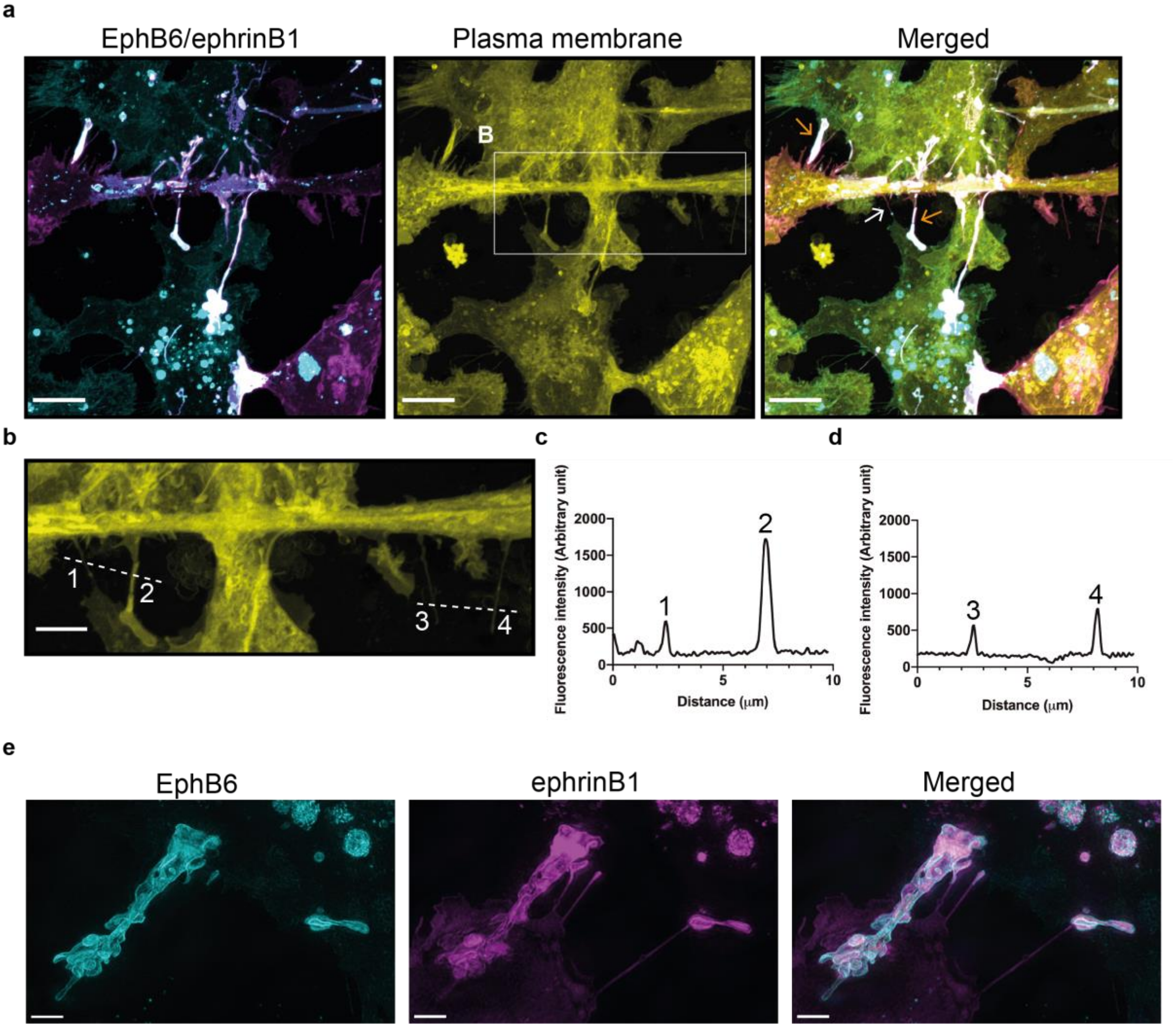

Supplementary Fig. 3: Characterization of the tubular structures decorated by the EphB6:ephrinB1 co-clusters, after chemical fixation of the co-cultured cells

a WT-EphB6-mNG (in cyan):ephrinB1-Halo (in magenta) co-cultured cells were fixed, stained with a plasma membrane dye (CellMask Orange) and imaged. The orange arrows indicate the tubular structures associated with the EphB6:ephrinB1 clusters. From a representative image, the white arrow indicates a tubular structure separate from the EphB6:ephrinB1 clusters. Images are presented as maximum intensity projections. The scale bar indicates 10 µm. **b** A zoomed-in image from Panel A, indicating the tubular and membrane protrusion structures, in which the fluorescence intensity was measured. The scale bar indicates 5 µm. **c** and **d** Measurement of the fluorescence intensity of the tubular structures and the membrane protrusions from Panel B. **e** The three-dimensional structured illumination microscopy (3D-SIM) images of the tubular structures decorated by the EphB6 (in cyan):ephrinB1 (in magenta) clusters, from chemically fixed cells. The scale bar indicates 2 µm.

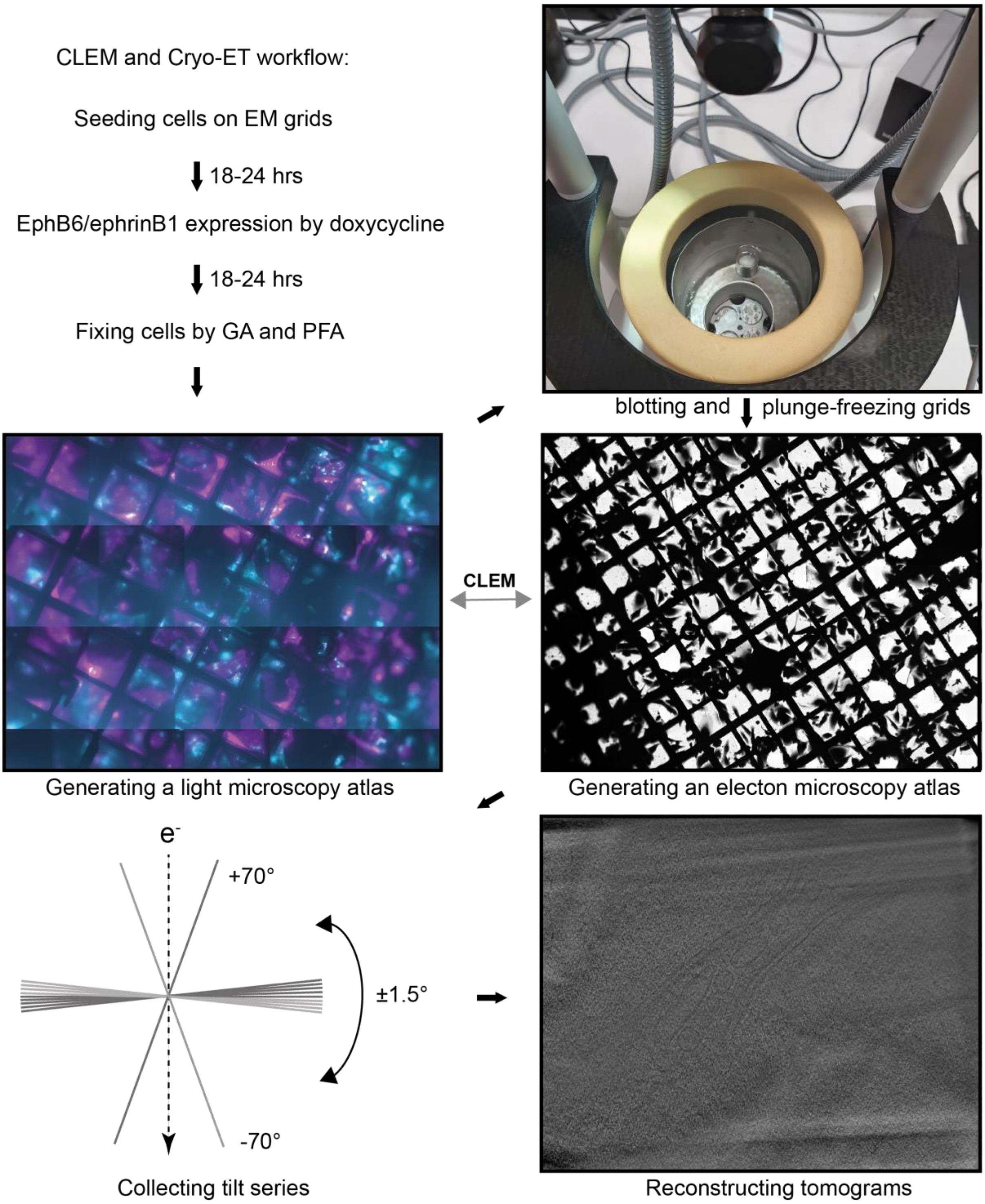

Supplementary Fig. 4: The CLEM and cryo-ET workflow

After cell seeding on the EM grids followed by doxycycline induction and chemical fixation, the EM grids were plunge-frozen and subjected to the CLEM workflow. GA: glutaraldehyde. PFA: paraformaldehyde. The whole EM grid was tile scanned by a Widefield microscope to generate a light microscopy atlas. The EM grids were then blotted and plunge-frozen in liquid ethane. After mounting the cryogenic EM grids on a transmission electron microscope, an

electron microscopy atlas was generated. The light and electron microscopy atlases were correlated (termed CLEM) based on the features of the grids, or the cell morphologies, which then allowed identification of the tubular structures decorated by the EphB6:ephrinB1 clusters for cryo-ET experiments. Cryo-ET was performed by collecting the tilt series at an increment of ± 1.5°, up to 70° in both rotations, followed by reconstruction of the tomograms using a procedure detailed in the STAR methods.

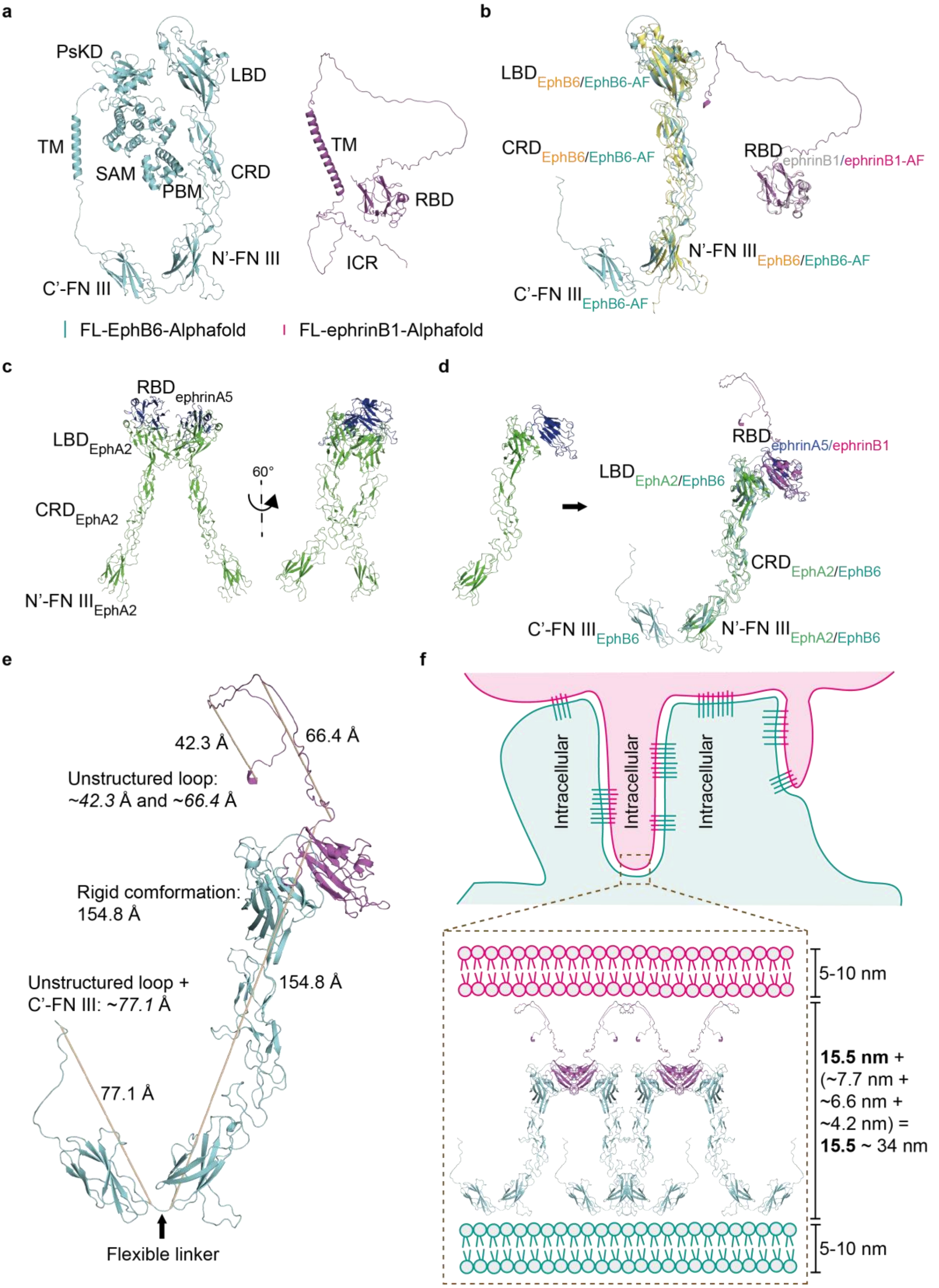
a
PsKD
LBD
TM
SAM
PBM
CRD
N'-FN III
C'-FN III
TM
RBD
ICR
FL-EphB6-Alphafold
FL-ephrinB1-Alphafold
b
LBD EphB6/EphB6-AF
CRD EphB6/EphB6-AF
RBD ephrinB1/ephrinB1-AF
N'-FN III EphB6/EphB6-AF
C'-FN III EphB6-AF
c
RBD ephrinA5
LBD EphA2
60°
CRD EphA2
N'-FN III EphA2
d
RBD ephrinA5/ephrinB1
LBD EphA2/EphB6
CRD EphA2/EphB6
C'-FN III EphB6
N'-FN III EphA2/EphB6
e
42.3 Å
66.4 Å
Unstructured loop:
~42.3 Å and ~66.4 Å
Rigid comformation:
154.8 Å
Unstructured loop +
C'-FN III: ~77.1 Å
154.8 Å
77.1 Å
Flexible linker
f
Intracellular
Intracellular
Intracellular
5-10 nm
15.5 nm +
(~7.7 nm +
~6.6 nm +
~4.2 nm) =
15.5 ~ 34 nm
5-10 nm

Supplementary Fig. 5: A structural model of the EphB6:ephrinB1 ectodomain protein complex fits the spacing between the double membranes of the tubular structures decorated by the EphB6:ephrinB1 clusters

a Structures of the full-length EphB6 (coloured in cyan) and ephrinB1 (coloured in magenta) predicted by AlphaFold. LBD: ligand-binding domain. CRD: cysteine-rich domain. N´-FN III: N-terminal fibronectin domain III. C´-FN III: C-terminal fibronectin domain III. TM: transmembrane domain. PsKD: pseudokinase domain. SAM: sterile α-motif domain. PBM: PDZ domain-binding motif. RBD: receptor-binding domain. ICR: intracellular region. **b** AlphaFold model of the complete extracellular components of EphB6 (Entry: O15197, residue 32-600, coloured in cyan) was superimposed on the experimentally determined structure of the EphB6 ectodomains (LBD + CRD + N´-FN III, PDB: 7K7J [1], coloured in yellow), rendering a RMSD of 3.323 Å. The AlphaFold structure of the complete extracellular components of ephrinB1 (Entry: P98172, residues 28-239, coloured in magenta) was superimposed on the experimentally determined structure of the ephrinB1 ectodomain (RBD, PDB: 6THG [2], coloured in grey), rendering a RMSD of 0.897 Å. **c** The experimentally determined structure of the hetero-tetrameric ectodomain complex of EphA2 (LBD + CRD + N'-FN III, coloured in green) and ephrinA5 (RBD, coloured in blue) (PDB: 3MX0 [3]) is presented. **d** The AlphaFold model of the complete EphB6 ectodomain (Entry: O15197, residues 32-600, coloured in cyan) and the AlphaFold model of the complete ephrinB1 ectodomain (Entry: P98172, residues 28-239, coloured in magenta) were superimposed on the structure of the hetero-dimeric complex of the EphA2 and ephrinA5 ectodomains (PDB: 3MX0 [3]), rendering a RMSD of 1.903 Å and a RMSD of 0.986 Å, respectively. **e** The approximate length of the extracellular components of EphB6 and ephrinB1 was measured in PyMOL. For EphB6, the length of the unstructured loop connecting the TM and the C´-FN III, together with the C´-FN III is roughly 77.1 Å. They are connected by a potentially flexible linker [4], to a more rigid configuration of the remaining ephrinB1 RBD-bound EphB6 ectodomains containing N´-FN III, CRD and LBD, which is 154.8 Å long. The ephrinB1 RBD is connected by an unstructured loop to its TM domain, with an estimated length of 66.4 Å + 42.3 Å, when it is in a partially extended conformation. **f** A proposed structure of the oligomeric ephrinB1-liganded EphB6 ectodomains is shown. The lipid bilayer consisting of a single plasma membrane is generally 5-10 nm long. Given the flexibility of the linker connecting the EphB6 N´-FN III and C´-FN III, and the unstructured loops of EphB6 and ephrinB1 that can potentially adopt a more extended or closed

conformation, the estimated length of the EphB6:ephrinB1 protein complex in the extracellular space is between 16 nm and 34 nm. The cartoons were made in PyMOL.

Supplementary Fig. 6: Evaluation of the EphB6 expression on the invasiveness of cancer cells

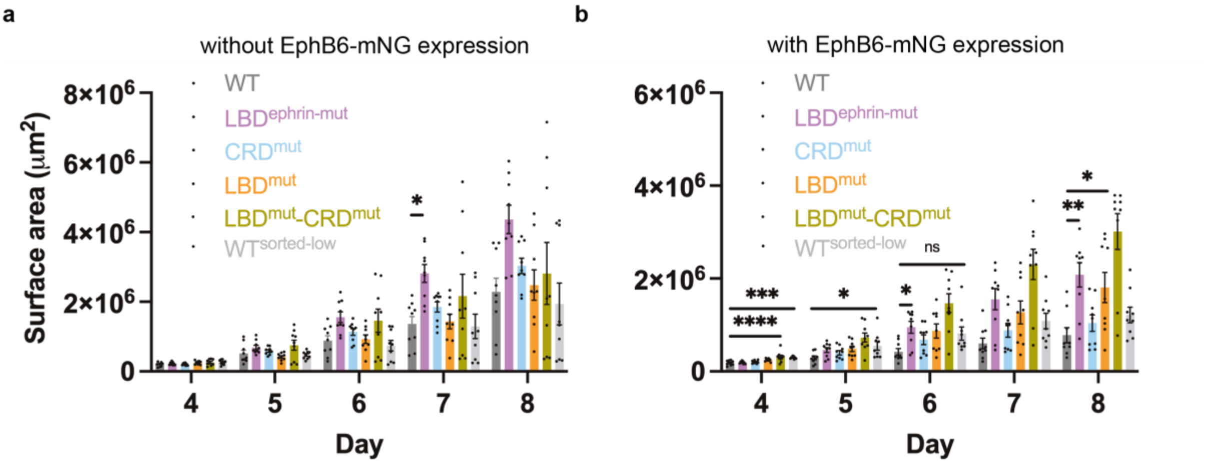

The invasiveness of the MDA-MB-231 cells is evaluated by measuring the area occupied by the MDA-MB-231 spheroids **a** without, and **b** with EphB6-mNG expression. N = total nine spheroids from each condition, from three biologically independent experiments. The data are represented by mean with ±SEM (standard error of the mean). Ns: no significance, *P < 0.05, **P < 0.01, ***P < 0.001 and ****P < 0.0001 were calculated by one-way ANOVA followed by a Dunnett's multiple comparison test.

1. Mason, E.O., et al., *Structure of the EphB6 receptor ectodomain.* PLoS One, 2021. **16**(3): p. e0247335.

2. Pryce, R., et al., *A key region of molecular specificity orchestrates unique ephrin-B1 utilization by Cedar virus.* Life Sci Alliance, 2020. **3**(1).

3. Himanen, J.P., et al., *Architecture of Eph receptor clusters.* Proc Natl Acad Sci U S A, 2010. **107**(24): p. 10860-5.

4. Xu, Y., et al., *The Ephb2 Receptor Uses Homotypic, Head-to-Tail Interactions within Its Ectodomain as an Autoinhibitory Control Mechanism.* Int J Mol Sci, 2021. **22**(19).

Supplementary Figure 7: Uncropped and unedited blot/gel images

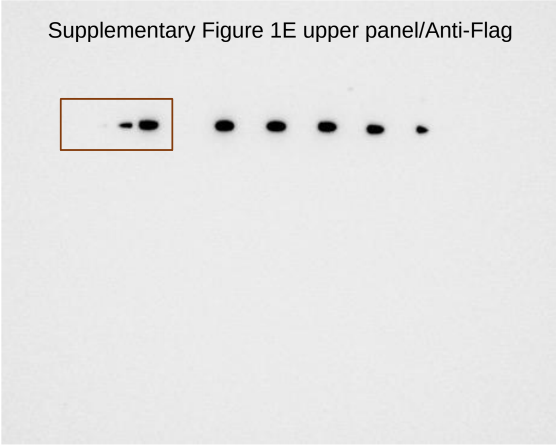

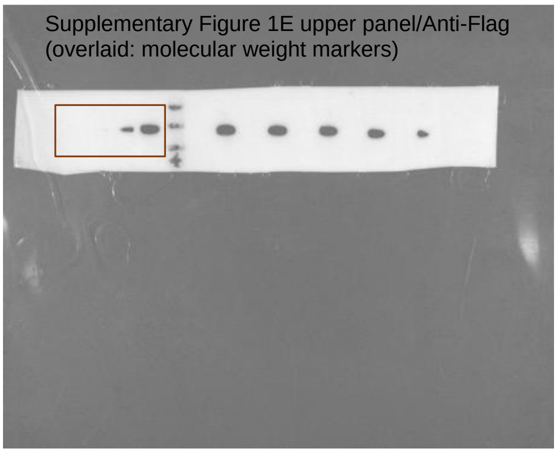

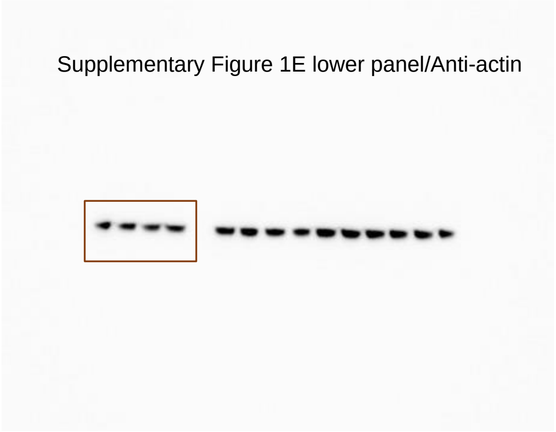

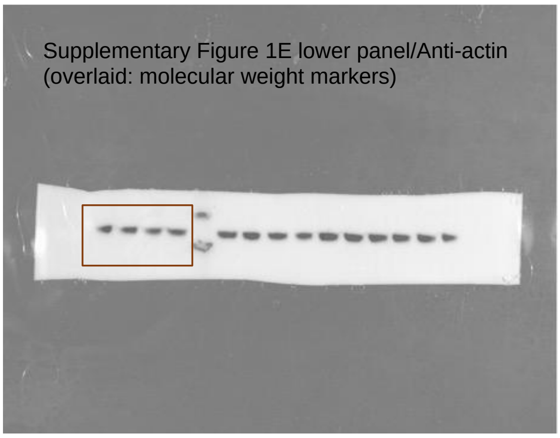

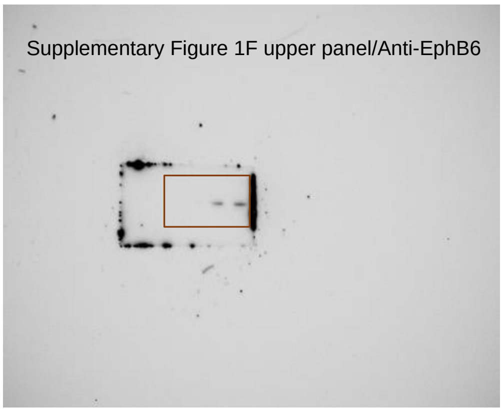

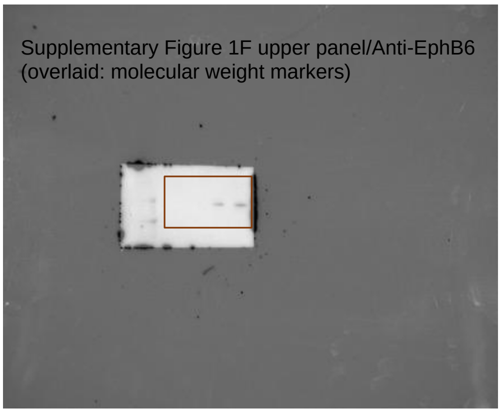

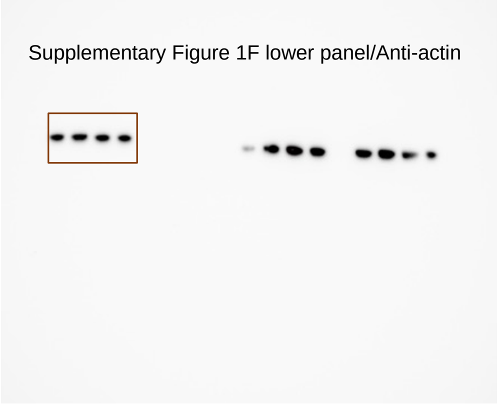

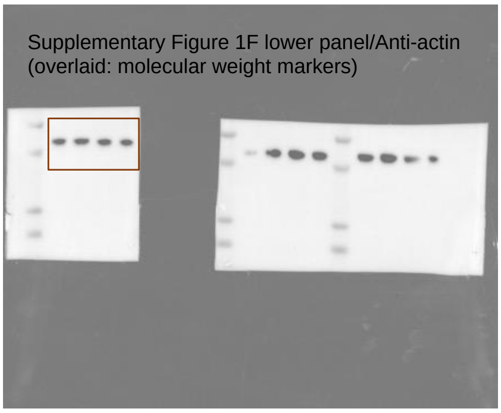

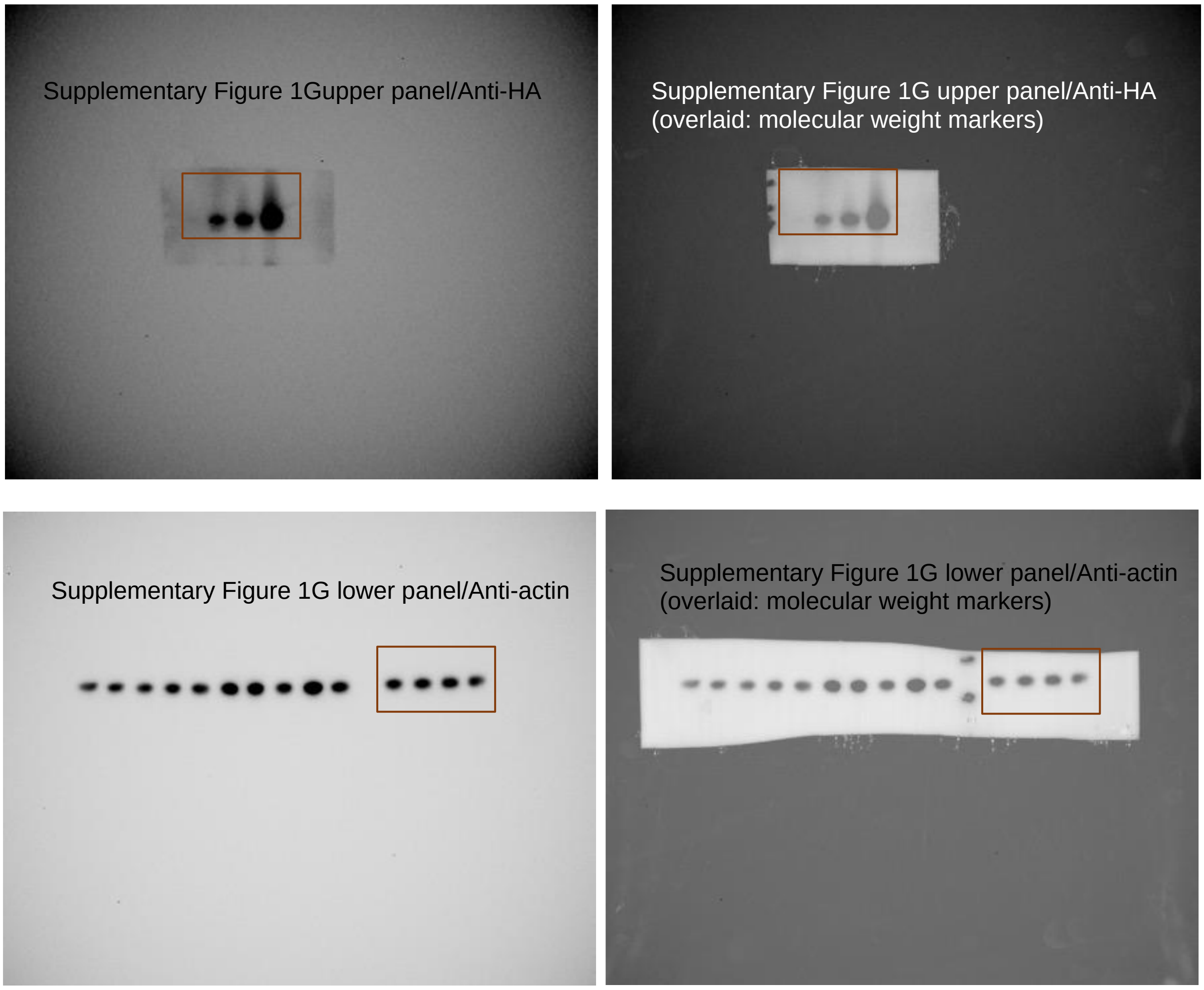
Supplementary Figure 1Gupper panel/Anti-HA
Supplementary Figure 1G upper panel/Anti-HA
(overlaid: molecular weight markers)
Supplementary Figure 1G lower panel/Anti-actin
Supplementary Figure 1G lower panel/Anti-actin
(overlaid: molecular weight markers)

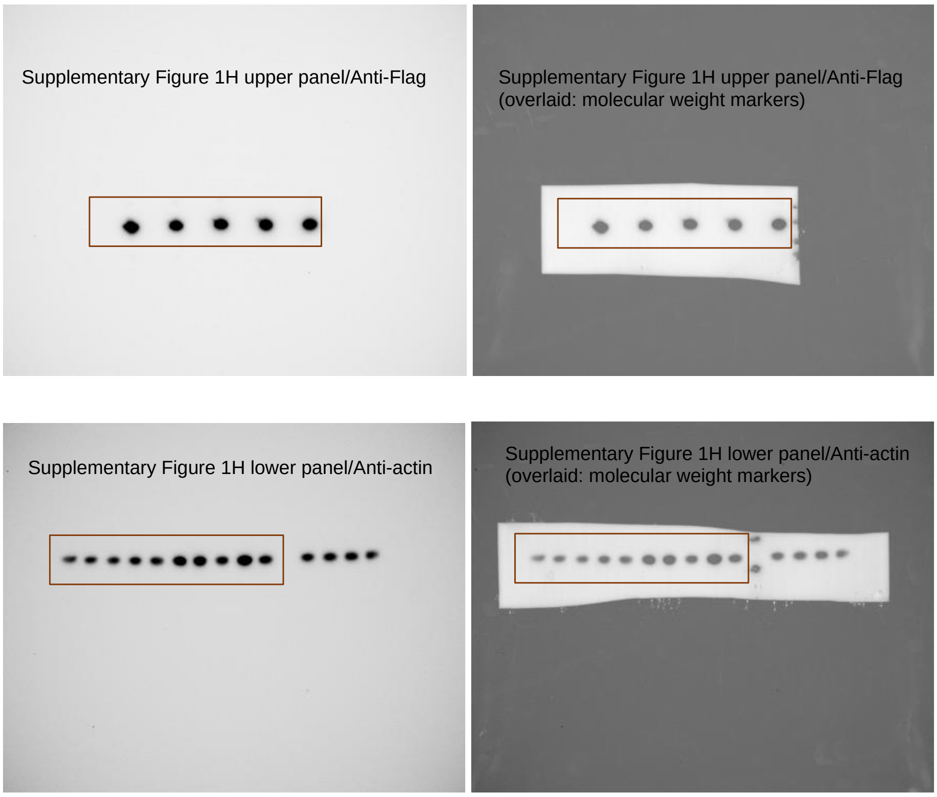
Supplementary Figure 1H upper panel/Anti-Flag
Supplementary Figure 1H upper panel/Anti-Flag
(overlaid: molecular weight markers)
Supplementary Figure 1H lower panel/Anti-actin
Supplementary Figure 1H lower panel/Anti-actin
(overlaid: molecular weight markers)

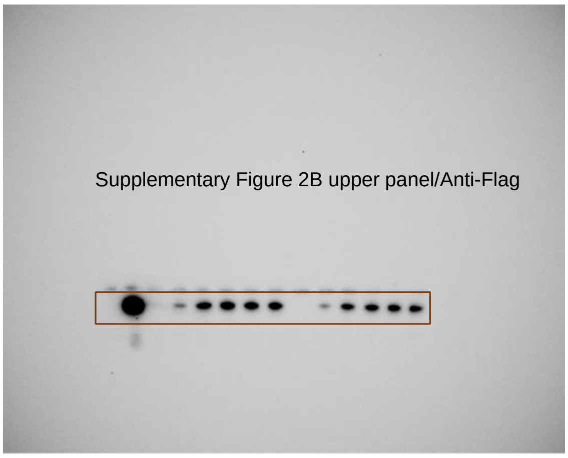
Supplementary Figure 2B upper panel/Anti-Flag

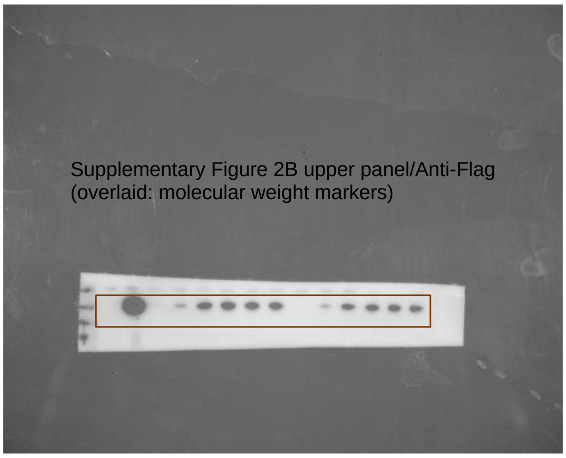
Supplementary Figure 2B upper panel/Anti-Flag
(overlaid: molecular weight markers)

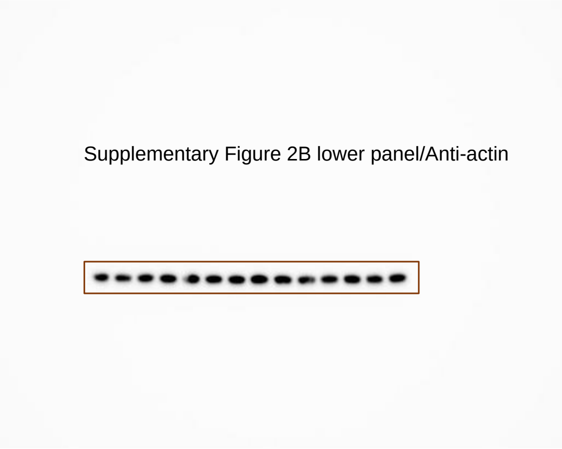
Supplementary Figure 2B lower panel/Anti-actin

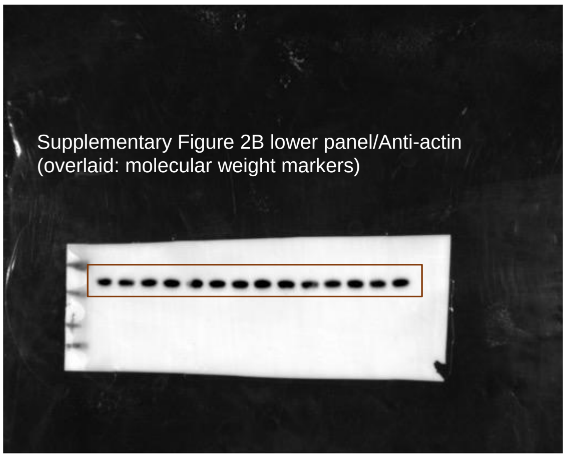
Supplementary Figure 2B lower panel/Anti-actin
(overlaid: molecular weight markers)

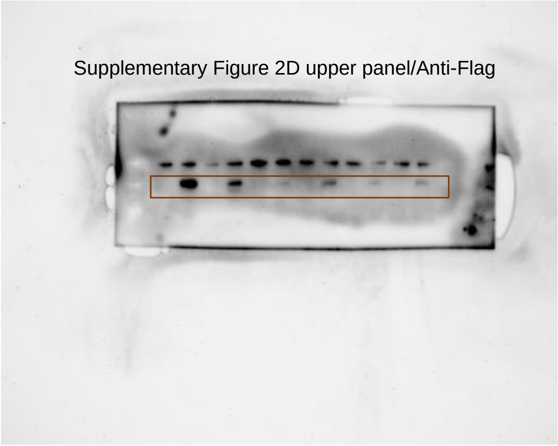
Supplementary Figure 2D upper panel/Anti-Flag

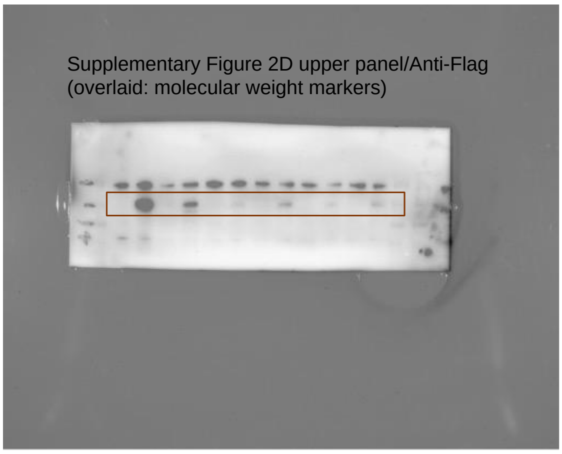
Supplementary Figure 2D upper panel/Anti-Flag
(overlaid: molecular weight markers)

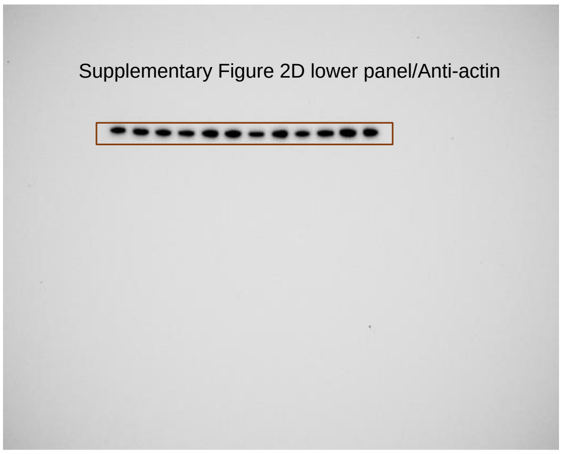
Supplementary Figure 2D lower panel/Anti-actin

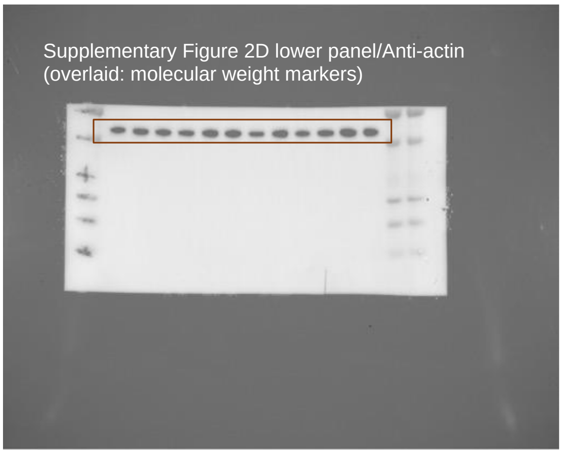
Supplementary Figure 2D lower panel/Anti-actin
(overlaid: molecular weight markers)
