# Supplementary material for: Co-clustering of EphB6 and ephrinB1 in trans restrains cancer cell invasion
Source: Commun Biol. 2024 Apr 16;7:461. doi: 10.1038/s42003-024-06118-4 (PMC11021433; doi:10.1038/s42003-024-06118-4)
Supplement: Supplementary file 2 — Description of Additional Supplementary Files [file 42003_2024_6118_MOESM2_ESM.pdf]

Description of Additional Supplementary Files

File name: Supplementary Data
Description: Numerical source data for Figures 1I, 2D, 3B, 5B, 5C, S6a, S6b.

File name: Supplementary Movie 1
Description: EphB6-ephrinB1 co-clustering and trafficking.

File name: Supplementary Movie 2
Description: WT-EphB1-ephrinB1 does undergo endocytosis.

File name: Supplementary Movie 3
Description: D744N-EphB1-ephrin B1 does undergo endocytosis.

File name: Supplementary Movie 4
Description: EphB6-ephrinB1 co-clusters exhibit a decreased propensity to endocytosis.

File name: Supplementary Movie 5
Description: Internalization of the EphB6:ephrinB1 co-clusters into EphB6-expressing cells captured by longer time-lapse image acquisitions.

File name: Supplementary Movie 6
Description: Unidirectional formation of the tubules with co-clusters – EphB6:ephrinB1 clusters first form when membrane protrusions of the ephrinB1 cells are in contact with the EphB6 cells.

File name: Supplementary Movie 7
Description: Formation of tubular structures occurs through co-clustering of EphB6:ephrinB1 between ephrinB1-HEK293 and EphB6-MDA-MB231 cells.

File name: Supplementary Movie 8
Description: Tomograms showing intracellular vesicles and filamentous structures resembling the cytoskeletal system present outside the double-membrane tubular structure.
