# Supplementary material for: Co-clustering of EphB6 and ephrinB1 in trans restrains cancer cell invasion
Source: Commun Biol. 2024 Apr 16;7:461. doi: 10.1038/s42003-024-06118-4 (PMC11021433; doi:10.1038/s42003-024-06118-4)
Supplement: Supplementary file 12 — Reporting summary [file 42003_2024_6118_MOESM12_ESM.pdf]

nature portfolio

Corresponding author(s): Isabelle Lucet
James Murphy

Last updated by author(s): 2024/03/15

Reporting Summary

Nature Portfolio wishes to improve the reproducibility of the work that we publish. This form provides structure for consistency and transparency in reporting. For further information on Nature Portfolio policies, see our Editorial Policies and the Editorial Policy Checklist.

Statistics

For all statistical analyses, confirm that the following items are present in the figure legend, table legend, main text, or Methods section.

n/a	Confirmed	
☐	☒	The exact sample size (*n*) for each experimental group/condition, given as a discrete number and unit of measurement
☐	☒	A statement on whether measurements were taken from distinct samples or whether the same sample was measured repeatedly
☒	☐	The statistical test(s) used AND whether they are one- or two-sided<br>*Only common tests should be described solely by name; describe more complex techniques in the Methods section.*
☒	☐	A description of all covariates tested
☒	☐	A description of any assumptions or corrections, such as tests of normality and adjustment for multiple comparisons
☐	☒	A full description of the statistical parameters including central tendency (e.g. means) or other basic estimates (e.g. regression coefficient) AND variation (e.g. standard deviation) or associated estimates of uncertainty (e.g. confidence intervals)
☒	☐	For null hypothesis testing, the test statistic (e.g. F, t, r) with confidence intervals, effect sizes, degrees of freedom and P value noted<br>*Give P values as exact values whenever suitable.*
☒	☐	For Bayesian analysis, information on the choice of priors and Markov chain Monte Carlo settings
☒	☐	For hierarchical and complex designs, identification of the appropriate level for tests and full reporting of outcomes
☒	☐	Estimates of effect sizes (e.g. Cohen's d, Pearson's r), indicating how they were calculated

Our web collection on statistics for biologists contains articles on many of the points above.

Software and code

Policy information about availability of computer code

Data collection

The confocal images were obtained by a Zeiss LSM980 Fast Airyscan 2 confocal microscope.
All lattice light-sheet microscopy experiments were performed on a Lattice Lightsheet 7 microscope (Zeiss – Pre-serial).
Super resolution three-dimensional structured illumination microscopy (3D-SIM) was performed on the DeltaVision OMX-SR system (GE Healthcare) equipped with a 60x/1.42 N.A. PlanApo oil immersion objective (Olympus), sCMOS cameras, and 488 and 568 nm lasers, and 1.516 refractive index immersion oil.
Correlative light and electron microscopy (CLEM) workflow: Cells fixed on the EM grids were imaged by a Zeiss Axio Observer Widefield microscope.
Cryo-ET was performed on a Titan Krios G4 cryo-transmission electron microscope (cryo-TEM) (ThermoFisher, US) operated at 300 keV. Images were acquired using the SerialEM software.
Spheroid invasion assays – image acquisition was performed using a widefield microscope (Zeiss Axio Observer)

Data analysis

Confocal images acquired were presented as maximum intensity projections in Fiji.
Lattice light-sheet microscopy: the acquired images were de-skewed and deconvolved using ZEN (Zeiss), and the post-processed images were presented as maximum intensity projections in Fiji.
Super resolution reconstruction and color channel alignment were performed with softWoRx 7.0 (GE Healthcare).
Cryo-ET: Tilt series were processed using IMOD (version 4.12). Mutual alignments of individual projections relied on the 10 nm gold fiducials using the IMOD software package. Back projection tomograms were generated using IMOD and simultaneous iterative reconstruction (SIRT) was then achieved using TOMO3D.
Spheroid invasion assays – image analysis. The resulting images were analyzed in Fiji/ImageJ.

All plots were generated in GraphPad Prism 9 (Dotmatics). All light microscopy data were analyzed in ImageJ (NIH) and Imaris x64 9.7.2 (Oxford Instruments).

For manuscripts utilizing custom algorithms or software that are central to the research but not yet described in published literature, software must be made available to editors and reviewers. We strongly encourage code deposition in a community repository (e.g. GitHub). See the Nature Portfolio guidelines for submitting code & software for further information.

Data

Policy information about availability of data

All manuscripts must include a data availability statement. This statement should provide the following information, where applicable:

- Accession codes, unique identifiers, or web links for publicly available datasets
- A description of any restrictions on data availability
- For clinical datasets or third party data, please ensure that the statement adheres to our policy

The datasets generated during and/or analysed during the current study are available from the corresponding author on reasonable request.

Human research participants

Policy information about studies involving human research participants and Sex and Gender in Research.

Reporting on sex and gender	N/A
Population characteristics	N/A
Recruitment	N/A
Ethics oversight	N/A

Note that full information on the approval of the study protocol must also be provided in the manuscript.

Field-specific reporting

Please select the one below that is the best fit for your research. If you are not sure, read the appropriate sections before making your selection.

☒ Life sciences ☐ Behavioural & social sciences ☐ Ecological, evolutionary & environmental sciences

For a reference copy of the document with all sections, see nature.com/documents/nr-reporting-summary-flat.pdf

Life sciences study design

All studies must disclose on these points even when the disclosure is negative.

Sample size	Sample size calculations were not performed. Sample sizes for each experiment are consistent with the field norms.
Data exclusions	The only data excluded in this study were from the Spheroid invasion assays. Where the spheroids were located outside the working distance of the microscope, their images were not acquired.
Replication	Experiments were performed at least twice as biological replicates; the actual number of replication was specified in the figure legends.
Randomization	Quantitation of the endocytosis rate of the Eph receptor clusters was performed on randomly selected area where the clusters can be discerned. Unbiased macros were applied to all experiments for quantitation.
Blinding	Selection and quantitation of the Eph receptor endocytosis rate were performed in a blinded manner.

Reporting for specific materials, systems and methods

We require information from authors about some types of materials, experimental systems and methods used in many studies. Here, indicate whether each material, system or method listed is relevant to your study. If you are not sure if a list item applies to your research, read the appropriate section before selecting a response.

Materials & experimental systems

n/a	Involved in the study	
☐	☒	Antibodies
☐	☒	Eukaryotic cell lines
☒	☐	Palaeontology and archaeology
☒	☐	Animals and other organisms
☒	☐	Clinical data
☒	☐	Dual use research of concern

Methods

n/a	Involved in the study	
☒	☐	ChIP-seq
☐	☒	Flow cytometry
☒	☐	MRI-based neuroimaging

Antibodies

Antibodies used	Mouse monoclonal anti-FLAG M2-HRP Sigma Cat# A8592; RRID:AB_439702<br>Goat polyclonal anti-mEphB6 R&D Cat# AF611; RRID:AB_2277844<br>Mouse monoclonal anti-β-actin-HRP Santa Cruz Cat# sc-47778 HRP; RRID:AB_2714189<br>Mouse monoclonal anti-pTyr (4G10) Millipore Cat# 05-321; RRID:AB_309678<br>Rat monoclonal anti-HA (3F10) Roche Cat# 11867423001; RRID:AB_390918<br>Sheep monoclonal anti-mouse-HRP GE Healthcare Cat# NA931; RRID:AB_772210<br>Donkey monoclonal anti-rabbit-HRP Amersham Cat# NA934; RRID:AB_772206<br>Mouse monoclonal anti-goat-HRP Santa Cruz Cat# sc-2354; RRID:AB_628490<br>Mouse monoclonal anti-FLAG M2 Affinity Agarose Sigma Cat# A2220; RRID:AB_10063035
Validation	For all antibodies, pecificity was validated as described on manufacturer's website. Anti-mEphB6 and Anti-Flag M2-HRP antibodies were also validated in this study as they only detected Flag tagged EphB6 upon doxycycline induction.

Eukaryotic cell lines

Policy information about cell lines and Sex and Gender in Research

Cell line source(s)	Human: HEK293T cells CRL-3216; RRID:CVCL_0063<br>Human: MDA-MB-231 cells HTB-26; RRID:CVCL_0062
Authentication	HEK293T cells were originally sourced from the American Tissue Culture Collection (ATCC), and MDA-MB-231 cells were provided by Dr Anderly Chueh (Walter and Eliza Hall Institute of Medical Research). MDA-MB-231 cells expressing exogenous EphB6, EphB1 or ephrinB1 were generated at the Walter and Eliza Hall Institute of Medical Research. None of the cell lines were genome sequenced. However, their morphologies are consistent with the stated cell type on the ATCC website.
Mycoplasma contamination	Wild-type HEK293T and MDA-MB-231 cells were tested mycoplasma negative. MDA-MB-231 cells expressing exogenous genes were tested mycoplasma negative when generated, and their growth rate remained normal during experimentation.
Commonly misidentified lines (See ICLAC register)	Nill

Flow Cytometry

Plots

Confirm that:

☐ The axis labels state the marker and fluorochrome used (e.g. CD4-FITC).

☒ The axis scales are clearly visible. Include numbers along axes only for bottom left plot of group (a 'group' is an analysis of identical markers).

☐ All plots are contour plots with outliers or pseudocolor plots.

☒ A numerical value for number of cells or percentage (with statistics) is provided.

Methodology

Sample preparation	Cells were induced by doxycycline for 16-24h prior to FACS analysis. Cells expressing mNeonGreen tagged EphB6 or EphB1 were analyzed.
Instrument	CytoFLEX Flow Cytometer (Beckman Coulter)
Software	FlowJo 10.1r7

Cell population abundance	Data were collected until 1000 events were recorded in the intact cell gate. Flow cytometry was used solely to quantify the number of cells based on the mNeonGreen intensities (at 525 nm).
Gating strategy	Cells were first gated to include only intact cells, based on forward and side scattering, and then cells were analyzed based on the mNeonGreen intensities (at 525nm).

☐ Tick this box to confirm that a figure exemplifying the gating strategy is provided in the Supplementary Information.
